# Experiencing COVID-19, home isolation and primary health care: A mixed-methods study

**DOI:** 10.3389/fpubh.2022.1023431

**Published:** 2023-01-10

**Authors:** Sandra Parisi, Nina Lehner, Hanna Schrader, Leonard Kierer, Anna Fleischer, Olga Miljukov, Gabor Borgulya, Gernot Rüter, Annika Viniol, Ildikó Gágyor

**Affiliations:** ^1^Department of General Practice, University Hospital Würzburg, Würzburg, Germany; ^2^Division of Medical Psychosomatics, University Hospital Würzburg, Würzburg, Germany; ^3^Institute of Clinical Epidemiology and Biometry, Julius Maximilian University of Würzburg, Würzburg, Germany; ^4^Academic Teaching Practice, Mentoring Team of the Competence-Based Continuing Education Baden-Württemberg Kompetenzzentrum Weiterbildung Baden-Württemberg (KWBW), University of Tübingen, Tübingen, Germany; ^5^Department of General Practice, University of Marburg, Marburg, Germany

**Keywords:** COVID-19, patients' experience, illness experience, mixed methods, general practice, home isolation, Germany, telehealth

## Abstract

**Objectives:**

Although the vast majority of COVID-19 cases are treated in primary care, patients' experiences during home isolation have been little studied. This study aimed to explore the experiences of patients with acute COVID-19 and to identify challenges after the initial adaptation of the German health system to the pandemic (after first infection wave from February to June 2020).

**Methods:**

A mixed-method convergent design was used to gain a holistic insight into patients experience. The study consisted of a cross-sectional survey, open survey answers and semi-structured telephone interviews. Descriptive analysis was performed on quantitative survey answers. Between group differences were calculated to explore changes after the first infection wave. Qualitative thematic analysis was conducted on open survey answers and interviews. The results were then compared within a triangulation protocol.

**Results:**

A total of 1100 participants from all German states were recruited by 145 general practitioners from August 2020 to April 2021, 42 additionally took part in qualitative interviews. Disease onset varied from February 2020 to April 2021. After the first infection wave, more participants were tested positive during the acute disease (88.8%; 95.2%; *P* < 0.001). Waiting times for tests (mean 4.5 days, SD 4.1; 2.7days, SD 2.6, *P* < 0.001) and test results (mean 2.4 days, SD 1.9; 1.8 days, SD 1.3, *P* < 0.001) decreased. Qualitative results indicated that the availability of repeated testing and antigen tests reduced insecurities, transmission and related guilt. Although personal consultations at general practices increased (6.8%; 15.5%, *P* < 0.001), telephone consultation remained the main mode of consultation (78.5%) and video remained insignificant (1.9%). The course of disease, the living situation and social surroundings during isolation, access to health care, personal resilience, spirituality and feelings of guilt and worries emerged as themes influencing the illness experience. Challenges were contact management and adequate provision of care during home isolation. A constant contact person within the health system helped against feelings of care deprivation, uncertainty and fear.

**Conclusions:**

Our study highlights that home isolation of individuals with COVID-19 requires a holistic approach that considers all aspects of patient care and effective coordination between different care providers.

## 1. Introduction

The novel coronavirus Sars-CoV2 has led to an unprecedented pandemic situation, affecting global economies, mobility and lives. As of November 2022, more than 6.6 million deaths have been attributed to COVID-19 (1). In addition to physical symptoms, COVID-19 leads to psychological distress and mental health consequences (2, 3). These can also be present in patients with non-severe COVID-19 (4, 5). Living through COVID-19 has been described as a stressful event and crisis (6).

The experience of people affected by COVID-19 could contribute to a holistic understanding of individuals‘ illness experience, but has (surprisingly) scarcely been studied (7). Most studies exploring experiences during the pandemic focus on non-infected individuals (8–10). The few studies exploring COVID-19 patients' experience often focus on vulnerable groups e.g., with comorbidities (11) or hospitalized patients (12–15). Both, comorbidities and hospitalization during COVID-19 may, however, significantly influence the illness experience (11–15). Hospital-based isolation is, for example, associated with post-traumatic stress disorder (PTSD) (16). While most patients experience a mild or moderate course of disease, little is known about the illness experience of the broader COVID-19 population in primary care.

During the course of the pandemic, health care systems had to prove resilience (17) finding new ways to ensure COVID-19 diagnosis and care, while adapting to hygiene, quarantine and isolation measures enforced to combat the spread of the disease. The adapted health structure included new modes of care and stakeholders: Telehealth services were rapidly expanded (18–20). In Germany, public health services (Öffentlicher Gesundheitsdienst) gained more influence and became a central player in patient management and implementing COVID-19 related policies (21) alongside general practitioners (GPs) who traditionally coordinate patient-centered prevention and care. Home isolation and care became an important element of the new health structure. While it has been postulated that isolation at home might be less burdensome than isolation in a hospital (22), home confinement during lockdowns has been shown to be a stressful event in the general population across countries (23). The influence of home isolation and the living situation during the pandemic on the individual illness experience is unknown (22). Moreover, there is a lack of evidence on how new modes of care, such as telehealth services, were perceived by patients, particularly within primary health care (24). The experience of people affected by COVID-19 with the new health care structures might help to identify remaining challenges and inform future policies.

Thus, the aim of this study was to explore the experiences of people affected by acute COVID-19 during diagnosis, course and care of disease. We also aimed to explore patients experience with the new health care structures and home isolation, as well as to identify remaining challenges after the initial adaptation of the health care system.

## 2. Materials and methods

### 2.1. Study design

We conducted a mixed-methods study consisting of a survey with both structured and open-ended questions and qualitative interviews. Mixed methods can combine the strength of both quantitative and qualitative research and add additional value when studying complex phenomena (25). We chose the design to gain a holistic insight into patients' experience, combining different methods to identify and compare emerging aspects. To increase credibility and validity of the results we used a large data set as well as methodological, data and investigator triangulation (11, 26).

**Figure 1:**
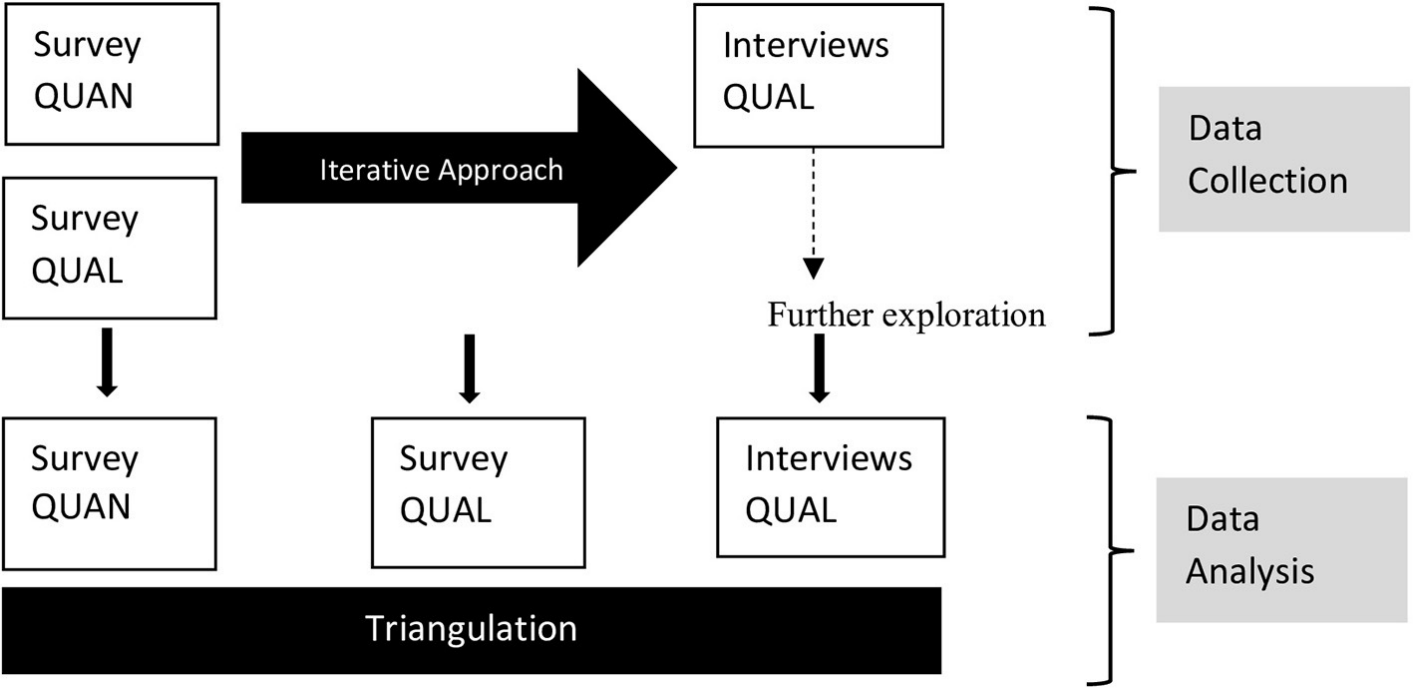
Mixed methods convergent design using parallel data collection with an iterative approach.

We used a convergent mixed-parallel design, collecting quantitative and qualitative data at the same time, with both methods equally weighted (27). The analysis of the different datasets was carried out independently and the results were then integrated for interpretation. We used an iterative approach (28) during data collection to fully explore aspects relevant to participants: Initial survey response and themes emerging within the open-ended survey questions were deepened within qualitative interviews (Figure 1).

The main findings arising within each component were then compared within a triangulation protocol (see also Supplementary material S1) for agreement, partial agreement, silence (complementing information, not present in all data sources) and disagreement (29, 30). This manuscript focusses on the experiences of patients during the acute disease whereas other issues we explored will be published elsewhere.

### 2.2. Study population and recruitment

Recruitment of patients took place through their GPs, who maintain the basic medical care for the population in the German health care system. GPs see the whole range of diseases courses and their patients holistically, within their lived reality during the COVID-19 pandemic.

All adult individuals who had ever experienced COVID-19 were eligible to participate. Due to the lack of access to diagnosis in the early phase of the pandemic, we also included patients without a positive PCR test, based on the early German case definitions (Robert Koch Institute (RKI) guidelines, Supplementary material S2). Exclusion criteria were living in a nursing home, alternative diagnosis (e.g., influenza) and not being able to fill out the survey by oneself (e.g., language constraints).

Study recruitment lasted from August 2020 to April 2021 to include the experience of participants during the so-called 2nd and 3rd infection waves in autumn and winter 20/21, when the German health care system had overcome its initial adaptation process to the pandemic situation. We invited GPs through various channels: a national online forum for GPs, practice based research networks, workshops and congresses, networks of GPs engaged in COVID-19 prevention. Moreover, we reached out to general practices in all German states through letters, using registered addresses publicly available on the internet. We estimate that we reached between 2.000–2.500 GPs in total.

**Figure 2:**
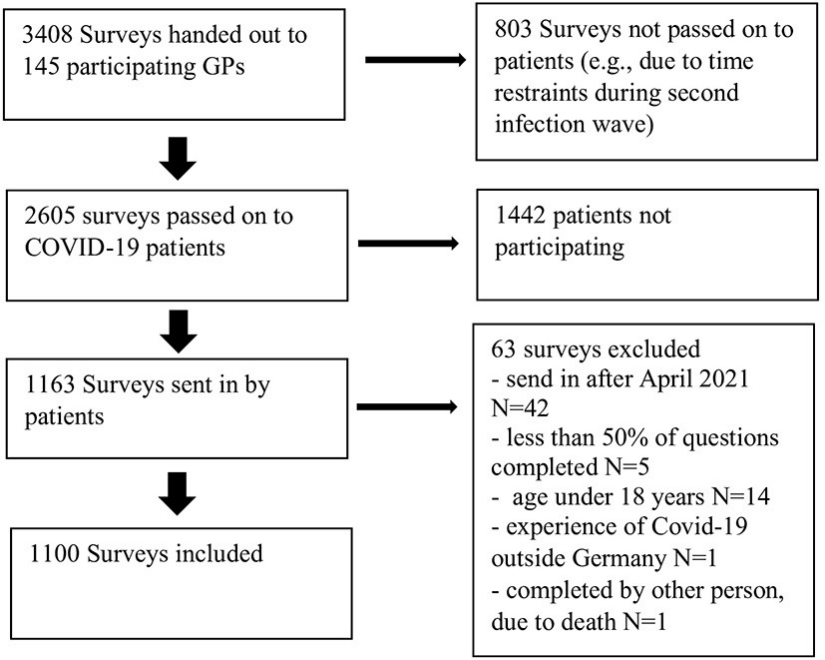
Flowchart of study recruitment.

Participating GPs were asked to contact all eligible patients and to pass along the study documents. The survey was then sent back directly to the department of General practice in Würzburg or filled out online using a link provided. Participants were asked to avoid any personal, identifiable information on the survey or envelops. Participants that additionally wanted to participate in telephone interviews directly contacted the study team. They received additional study information including informed consent. Qualitative sampling was by convenience, including all participants until a broad range of participants and illness experiences was covered, and no new themes emerged (data saturation). After finalization of recruitment, GPs were contacted to assess how many study documents they had effectively passed along to their patients for a clearer estimate of the response rate (Figure 2).

All documents and tools were tested and adapted during a pilot phase in a community of Würzburg in June/July 2020. A total of 15 patients recruited by 1 non-participating GP completed the survey during the pilot phase. Survey responses were checked and adapted in detail for consistency and misunderstandings. The participants provided written feedback on the survey structure, length and comprehensibility of questions and were asked to propose better suiting answers if appropriate.

### 2.3. Data collection and analysis

#### 2.3.1. Quantitative data

The 52-items survey (Supplementary material S3) included sociodemographic variables, comorbidities and risk factors for severe COVID-19, alongside questions on the course and duration of symptoms, as well as on patients' experience during the process of diagnosis and care. Several questions were partially categorized, with the option of multiple choices and the option “others” that could then be specified. Partially categorized questions enable simplified data collection by easier coding of frequently expected answers, without forcing divergent answers into pre-defined categories and thus distorting the results (31). They can moreover help to identify additional aspects relevant to the patients' experience.

Paper-based survey data was entered manually into REDCap electronic data capture tools hosted at [University of Würzburg] (32) with 10% of surveys being randomly selected for independent double data entry (NL, SP) to rule out systematic errors. Both, paper and online databases were then imported into SPSS (IBM Corp. Released 2020. IBM SPSS Statistics for Windows, Version 27.0. Armonk, NY: IBM Corp) and merged. Respondents that did not meet the eligibility criteria and surveys with < 50% of questions answered, were excluded.

Descriptive analysis of all variables, graphs and bar charts served for database exploration using (33, 34). Comments specified within the option “other” in partially categorized questions were either categorized or explored through qualitative methods. To assess differences in experiences and to identify remaining challenges after the initial adaptation of the health care system to the pandemic, a dichotomous variable for the first infection wave was created (0 = first infection wave, 1 = after first wave). We used the cutoff date for the first infection wave specified in the official reports from RKI (35) (including reporting week 25/20, 21st June 2020). Between group differences were calculated using Kruskal-Wallis (ordinal variables), *t*-tests, Chi Square or Fisher's exact tests, where appropriate.

#### 2.3.2. Qualitative data

Qualitative survey data included responses written within free spaces for comments on the experience during the diagnostic process and any relevant aspects related to the illness experience. Participants also added written side notes or used the option “other” in the section of illness and care for longer comments, e.g., related to health care deprivation. We decided to include those comments into qualitative thematic analysis.

Interviews were conducted *via* telephone, audio-recorded and transcribed verbatim using a semi-structured interview guide (Supplementary material S3), which was adapted during the process according to new or saturated themes. Thematic analysis with a content-structuring approach aligned to Kuckartz (36) was performed in MAXQDA 2020 (VERBI Software 2019) (37). Main categories were identified deductive-inductively on the ground of the interview guideline and study objectives. After coding all transcripts into those main categories, sub-categories were identified inductively. Two researchers (NL, LK, both thesis students) conducted and coded approximately half of the interviews. SP (MD, MScIH, experienced in mixed-methods research) then built a final coding structure by merging both frameworks and re-coding the data using consensual coding and discussing differences within the research team. IG (MD, senior researcher) supervised the process.

#### 2.3.3. Triangulation of results

Key findings were identified within each data set and listed within a triangulation protocol (S1). Quantitative findings were reframed into qualitative statements for comparison. Three researchers then independently compared those key findings for agreement, partial agreement, silence or disagreement (SP, NL, KL). Differences were resolved by discussion.

### 2.4. Public involvement

The idea for the study emerged within an online forum of German GPs (Allgemeinmedizinischer Listserver) (38) and was then elaborated by the research team and a member of the forum (GR). Both GPs and patients were involved in the initial design and adaptation of the study and its tools. Several forums were used to engage GPs and patients in this process: (1) Patients gave written and oral feedback on the tools and missing topics they considered important before and after the pilot phase. (2) Initial results were discussed and compared to experiences of local GPs during a workshop. (3) Some authors had also their own experiences with COVID-19.

### 2.5. Ethics

The study was approved by the ethical committee of the university hospital Würzburg (Nr. 135/20 opinion dated 24th of June 2020). The survey was anonymous. Written informed consent was provided by all participants of qualitative interviews.

## 3. Results

**Figure 3:**
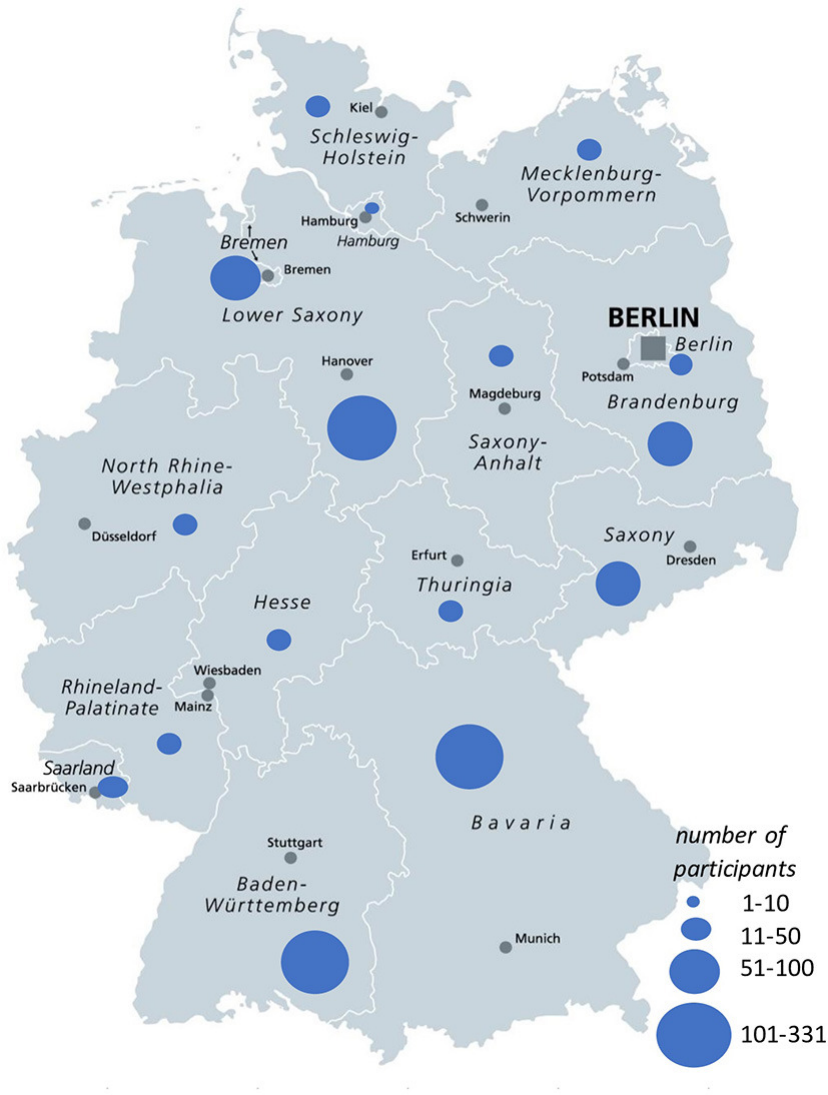
Distribution of survey participants per federal state. Adapted by NL from Peter Hermes Furian–shutterstock.com, with permission.

A total of 145 GPs supported the study by handing out 2605 surveys to their respective patients from August 2020 to April 2021. From 1163 completed surveys, 1100 were included in the analysis, (90.8% paper based, 9.2% online). The response rate was 44.6%. Figure 2 depicts the flowchart of study recruitment. Figure 3 shows the distribution of survey participants per federal state.

### 3.1. Sociodemographic characteristics of the study population

Survey participants were aged 18–93 years (mean 51.0, SD 16.0, median 53.0) and 56.9% were female; 21.5% worked in health care. Disease onset varied between February 2020 and April 2021. Table 1 depicts the main characteristics of survey participants. Of these, 42 participants (26 female) attended qualitative interviews *via* telephone (detailed description of interview participants in Supplementary material S4).

**Table 1:** Sociodemographic characteristics of the survey population.

**Variable**	**Categories**	**Total (%) *N* = 1,100**
Age	18–29	149 (13.5)
	30–39	114 (10.4)
	40–49	197 (17.9)
	50–59	300 (27.3)
	60–69	216 (19.6)
	70–79	79 (7.2)
	80–93	40 (3.6)
Gender	Female	627 (57.0)
	Male	468 (42.5)
	Diverse	1 (0.1)
Education	Incomplete schooling	20 (1.8)
	Secondary comprehensive	272 (24.7)
	Secondary	333 (30.3)
	Grammar	164 (14.9)
	Academic degree	293 (26.6)
Work status	Employed full time	523 (47.5)
	Employed part time	225 (20.5)
	Self–employed	63 (5.7)
	Minijob^*^	32 (2.9)
	Job seeking	9 (0.8)
	Not working^**^	238 (21.5)
Working in health care		236 (21.5)
Vulnerable household member		295 (26.8)
Underage household member		295 (26.8)
Existential financial insecurities due to the pandemic		144 (13.1)
History of psychiatric disease		127 (11.5)
History of lung disease	Asthma	89 (8.1)
	COPD	19 (1.7)
	Pneumonia (last 5 years)	37 (3.4)
	Other lung disease	42 (3.8)
Cardiovascular risk factors	Hypertension	296 (26.9)
	Other cardiovascular disease	98 (8.9)
	Diabetes	85 (7.7)
	Adipositas per magna (BMI > 40)	57 (5.2)
	Smoker	110 (10.0)
Other preexisting medical conditions with potential relation to COVID−19	Oncologic disease	47 (4.3)
	Immunological disease	39 (3.5)
	Disease requiring immunosuppressives	21 (1.9)
	Liver disease	16 (1.5)
	Kidney disease	25 (2.3)
	Other	106 (9.6)

* Working contract exempted from taxes with a maximum of 450 Euro or 70 working days/year;

** e.g., retired, student, maternal/paternal leave.

Missing Values: Age N = 5 (0.5%), Gender N = 4 (0.4%), Education N = 18 (1.6%), Work situation N = 12 (1.1%), Work in HC N = 17 (1.5%), Living with vulnerable household member N = 11 (1.0%), Underage household member N = 3 (0.3%), Existential financial insecurities due to the pandemic N = 10 (0.9%), smoking habit N = 7 (0.6%), History of psychiatric disease missing N = 6 (0.5%), don't know N = 25 (2.3), don't want to answer N = 15 (1.4%).

### 3.2. Experiences during the diagnostic process of COVID-19

Most participants (79.7%) had actively sought diagnosis, mainly due to the occurrence of symptoms (52.8%) and the majority had received a diagnostic test (95.5%), with 93.3% of these being positive. Every fifth participant had later undergone serologic testing, with 78.4% being positive. After the first infection wave there were significantly more positive test results (*P* < 0.001). There was a decrease in the time span between developing symptoms and tests (*P* < 0.001) and waiting for test results (*P* < 0.001) (Table 2).

**Table 2:** Experiences of people affected by acute COVID−19, summary of quantitative results.

**A) Experiences during the diagnostic process**					
**Variable**	**Categories**	**Total (%)**	**1st wave (%)**	**After 1st wave**	***P*** **–value**
		***N** =* **1,100**	***N** =* **237**	***N** =* **799**	
Active diagnosis seeking		877 (79.7)	181 (77.7)	657 (83.7)	**0.035**
Reasons for seeking diagnosis	Presence of symptoms	586 (53.3)	116 (48.9)	443 (55.4)	0.078
	Worried to be infected	400 (36.4)	86 (36.3)	296 (37.0)	0.831
	Relatives belonging to risk group	203 (18.5)	43 (18.1)	151 (18.9)	0.794
	Part of a scientific study	0 (0)	0(0)	0(0)	
	Professional reasons (e.g., HCW)	225 (20.5)	40 (16.9)	174 (21.8)	0.102
	Asked to get tested	295 (26.8)	65 (27.4)	215 (26.9)	0.875
	To shorten the quarantine	27 (2.5)	6 (2.5)	21 (2.6)	0.935
	Contact with a positive person	84 (7.6)	13 (6.3)	67 (9.3)	0.168
	Other	71 (6.5)	27 (11.4)	40 (5.0)	**< 0.001**
Serologic test after acute disease		233 (21.1)	106 (45.1)	120 (15.3)	**< 0.001**
Positive serologic test result		181 (78.4)	90 (84.9)	86 (72.9)	**0.029**
Diagnostic test during acute disease		1051 (95.5)	224 (94.5)	765 (95.7)	0.144
Positive test result during acute disease^*^		981 (93.3)	199 (88.8)	729 (95.2)	**< 0.001**
Perception time span between symptoms and test^*^	Very short	211 (20.1)	27 (12.1)	173 (22.6)	**< 0.001**
	Short	247 (23.5)	49 (21.9)	187 (24.4)	
	Adequate	316 (30.1)	66 (29.5)	230 (30.1)	
	A little too long	99 (9.4)	26 (11.6)	64 (8.4)	
	Much too long	70 (6.7)	39 (17.4)	30 (3.9)	
Perceived duration till test result^*^	Very short	157 (14.9)	33 (14.7)	114 (14.9)	**< 0.001**
	Short	245 (23.3)	56 (25.0)	176 (23.3)	
	Adequate	365 (34.7)	66 (29.5)	278 (36.3)	
	A little too long	163 (15.4)	37 (16.5)	112 (14.6)	
	Much too long	107 (10.2)	28 (12.5)	76 (9.9)	
Place of first test^*^	General practice	635 (60.4)	78 (34.8)	520 (68.0)	**< 0.001**
	Public health department	39 (3.7)	13 (5.8)	22 (2.9)	**0.038**
	Emergency unit	31 (2.9)	15 (6.7)	14 (1.8)	**< 0.001**
	Test center	198 (18.8)	75 (33.5)	114 (14.9)	**< 0.001**
	Occupational physician	13 (1.2)	2 (0.9)	10 (1.3)	1.000
	Specialist (e.g., respiratory)	7 (0.7)	0	7 (0.9)	0.361
	At home	64 (6.1)	30 (13.4)	31 (4.1)	**< 0.001**
	Others	56 (5.3)	11 (4.9)	43 (5.6)	0.669
Days between initial symptoms and first test		3.1 (3.1)	4.5 (4.1)	2.7 (2.6)	**< 0.001**
Days between test and receiving test result		2.0 (1.5)	2.4 (1.9)	1.8 (1.3)	**< 0.001**
**B) The COVID–illness experience**					
**Variable**	**Categories**	**Total (%)**	**1st wave (%)**	**After 1st wave**	***P*** **-value**
		***N** =* **1,100**	***N** =* **237**	**(%)** ***N** =* **799**	
Course of disease	Pneumonia	85 (7.7)	30 (12.7)	49 (6.2)	**< 0.001**
	Hospital Admission	103 (9.4)	38 (16.0)	59 (7.4)	**< 0.001**
	Oxygen Suppl.	74 (6.7)	26 (11.6)	44 (5.8)	**0.003**
	Intensive Care	26 (2.4)	10 (4.2)	16 (2.0)	0.056
	Ventilation	18 (1.6)	6 (2.6)	10 (1.3)	0.224
Prescribed medication	No medication	654 (59.5)	140 (59.1)	477 (59.7)	0.863
	Antipyretics/NSAIDS	297 (27.0)	66 (27.8)	215 (26.9)	0.775
	Antibiotics	110 (10.0)	34 (14.3)	72 (9.0)	**0.017**
	Antivirals	12 (1.1)	2 (0.8)	10 (1.3)	1.000
	Phytotherapy	51 (4.6)	6 (2.5)	42 (5.3)	0.080
	Homeopathy	26 (2.4)	5 (2.1)	19 (2.4)	0.809
	Other	143 (13.0)	22 (9.3)	108 (13.5)	0.084
Self–medication	No medication	349 (31.7)	94 (39.7)	231 (28.9)	**0.002**
	Antipyretics/NSAIDS	538 (48.9)	94 (39.7)	414 (51.8)	**0.001**
	Antibiotics	21 (1.9)	2 (0.8)	17 (2.1)	0.273
	Antivirals	13 (1.2)	2 (0.8)	9 (1.1)	1.000
	Phytotherapy	161 (14.6)	26 (11.0)	128 (16.0)	0.055
	Homeopathy	82 (7.5)	13 (5.5)	65 (8.1)	0.175
	Other	178 (16.2)	31 (13.1)	140 (17.5)	0.106
Care provider during COVID−19/	GP	762 (69.3)	169 (71.3)	548 (68.6)	0.425
	Infectious disease consultation	40 (3.6)	3 (1.3)	34 (4.3)	**0.028**
	Infectious disease ambulance	12 (1.1)	2 (0.8)	8 (1.0)	1.000
	Specialist (e.g., respiratory)	29 (2.6)	8 (3.4)	21 (2.6)	0.540
	Public Health department	511 (46.5)	109 (46.0)	377 (47.2)	0.747
	Other	173 (15.7)	45 (19.0)	121 (15.1)	0.157
Care mode	Telephone	862 (78.4)	192 (81.0)	627 (78.5)	0.399
	Normal consultation	150 (13.6)	16 (6.8)	124 (15.5)	**< 0.001**
	Video consultation	17 (1.5)	2 (0.8)	15 (1.9)	0.387
	Other	175 (15.9)	38 (16.0)	126 (15.8)	0.922

* N total = 1,051, N first wave (disease onset until 21st of June 2020) = 224, N after first wave = 765 (N total = participants tested).

Missing date of disease onset: N = 64 (5.8%); **Missing diagnostic process:** Active seeking of diagnosis? N = 24 (2.2%), don't know N = 13 (1.2%), Was a PCR Test taken during acute disease? N = 2 (0.2%) Don't know N = 1 (0.1%), No N = 46 (4.2%), Test result missing N = 30 (2.9%), Don't know N = 7 (0.7%), Perception on time span between symptoms and test? N = 108 (10.3%). Perception time span results N = 15 (1.4%), Place of first test missing N = 8 (0.8%), Serologic test N = 20 (1.8%), Don‘t know N = 30 (2.7%), Serologic test result missing N = 2 (0.2%) **Missing illness experience:** Pneumonia N = 6 (0.5%), Hospital Admission N = 4 (0.4), Intensive Care N = 6 (0.5), Ventilation N = 8 (0.7%), Oxygen Supply N = 53 (4.8%).

Bold values indicate P-Values < 0.05 that are considered statistically significant.

#### 3.2.1. Experiences during the diagnostic process–qualitative survey results

Accessing diagnosis ranged from smooth testing (VS1) to nerve-wrecking experiences (VS2-4). Reasons for not being tested were not complying with test criteria (e.g., high fever, positive contact, work within health care) (VS5-7), misdiagnosis (VS8), denial by health authorities and GPs (VS9-11), prioritization due to the scarcity of tests (VS12-14) and problems getting to the test site while feeling sick (VS15-16). Dedicated GPs, focal points for COVID-19 and working within healthcare were facilitators for early testing and early information of results, even outside of working hours (VS17-21). Referenced verbatims (VS) can be found in Supplementary materials S5, S6.

Long waiting times for test results led to insecurities among participants (VS22-23), household members, contacts and superiors (VS24-26) and were connected to putting other people unnecessarily at risk (VS27-28) and to the ongoing high Sars-CoV2 incidence (VS29). Later, the availability of antigen tests and possibilities to repeated testing decreased these insecurities (VS30-32).

#### 3.2.2. Experiences during the diagnostic process–qualitative interviews

The efforts to obtain diagnosis and access barriers were similar to qualitative survey answers (VS33-36). Some participants highlighted creative solutions among their GPs to assure early diagnosis (VS37-39), while others perceived lack of expertise (VS40) or felt denied diagnosis (VS41). Participants also stressed the logistic tasks associated with COVID-19 (VS42) and difficulties organizing the diagnosis of household members (VS43) and contacts (VS44). Public authorities were often perceived overstrained (VS45-46), with difficulties in managing early diagnosis and transmission control, also due to the lack of coordination and digitalization (VS47-49).

The emotional reaction to being diagnosed with COVID-19 ranged from disbelief (VS50-51) and shock (VSVS52-53) to staying calm (VS54) or feeling relief of finally being sure about the diagnosis (VS55). Diagnosis led to rumination and distress about prior contact behavior (VS56-58).

### 3.3. The COVID-19 illness experience

The most cited symptoms of acute COVID-19 were fatigue (70.3%), body aches (60.8%), headaches (60.4%), cough (57.9%), dysosmia (55.7%) and dysgeusia (53.6%) being present in more than half of the study population (Supplementary material S7). Participants with COVID-19 experience during the first wave, more often reported a severe course of disease (Table 2).

GPs (69.3 %) and the public health department (46.5%) were cited as the main care providers. While diagnosis and personal consultations at general practices increased after the first wave (6.8%; 15.5%, *P* < 0.001), telephone consultation remained (78.5%) the main mode of care and video consultation remained insignificant (1.9%). Some participants (6.1% of total study population) emphasized within the option “other” that they had received no or inadequate health care, without a significant decrease after the first infection wave (*P* = 0.571). The option “other” also included privately organized or complementary care. Survey responses on prescribed and self-medication are depicted in Table 2.

#### 3.3.1. Illness experience- qualitative survey results

Disease progression and symptoms: Participants reported a wide range of disease severity (VS 59-61). Severe courses of disease, leading to hospitalization (VS62), but also the unexpected intensity and duration of common symptoms such as pain (VS63-64), fever (VS62), cough (VS65) and especially weakness (VS60, 66-67) impaired the participants‘ illness experience. The unpredictability of COVID-19, with sudden changes in symptoms and severity and the undulating recovery process was perceived difficult (VS63, 68-69).

The mental burden caused by COVID-19: Several participants described panic attacks and constant fear (VS70-72). The focus of media and politicians on severe COVID-19 (VS73) and belonging to a risk group (VS74) contributed to the mental burden. Isolation led to problems with caring for vulnerable family member and worries (VS75-76). Participants described loneliness, imprisonment and difficulties coping with their symptoms, especially when living alone (VS75-77).

Access to health care was often described difficult, both in person and by phone (VS78-80). Perceived health care deprivation during isolation led to feelings of being left alone (VS81-83), helplessness (VS83-84) and insecure (VS85). Insecurities also emerged from the perceived lack of expertise among health staff (VS86-87). A lack of coordination among health care providers resulted in contradicting information and increased the organizational burden on participants (VS88-91). There were, however, also participants with positive experiences (VS92) and participants who reported no need for further care (VS93). Moreover, several participants highlighted their family practice as supporting pillar (VS94-95). Other identified themes are summarized in Table 3.

**Table 3:** Experiences of people affected by acute COVID−19, summary of qualitative results.

**A) Experiences during the diagnostic process**	
**Survey QUAL**	**Interviews QUAL**
• Accessing a test - Reasons for not having been tested. - Individual effort of care providers. • Waiting for test results - Feeling tense and anxious. - Difficulties interrupting transmission chains. - Later availability of antigen tests and multiple testing.	• Accessing diagnosis - Effort to obtain diagnosis. - Access barriers. - Creative solutions by individual GPs. - Long waiting time for test results. - Experiencing ambiguous/unclear results. - Diagnosis of household member. • Emotional response - Disbelief of having caught COVID−19. - Shock and fear. - Taking it calm. - Relief through certainty.
**B) The COVID**−**19 illness experience**	
**Survey QUAL**	**Interviews QUAL**
• Disease progression and symptoms - From harmless to worst disease ever. - Unprecedented severity of common symptoms. - Description of rare symptoms. - Unpredictable dynamic of illness. • Mental burden caused by COVID−19 - Panic and fear of death. - Scaremongering of media and politics. - Loss of joie de vivre due to loss of taste and smell. - Loneliness and Imprisonment during isolation. • Access to health care - Health care deprivation during isolation. - Insecurities caused by lack of organization within the health system. - Wide range of experience with health authorities. - No need and privately organized care. - General practitioners as supporting pillar. • Influence of spirituality and view on life - Faith in God. - Staying positive. • Influence of social surroundings - Fear of infecting others. - Worries for family member. - Burden caused by death of others. - Perception of Stigma.	• Disease course and progression - Wide range of severity. - Uncertain course of disease. - Increased body alertness. - Debilitating symptoms and their duration. - Fear of severe course and death. - Decompensation of comorbidities. - Hospitalization and ICU admission. • Living situation during isolation - Imprisonment: Available space and Access to garden. - Distraction. - Social surroundings. - A household full of patients. - Loneliness. • Access to health care - Health deprivation during isolation. - No need for care or personally organized care. - Bureaucracy and lack of organization. - A constant contact person vs. anonymity. • Personal strength and resilience - Physical and mental comorbidities. - Dealing with information overload. - Being a positive person. • Feelings of guilt and worries - Transmission before knowing one's diagnosis. - Disease of family member.

#### 3.3.2. Illness experience–qualitative interviews

Disease course and progression: The intensity (VS96-99), duration (VS99-101) and combination (VS100-101) of pain, weakness, fever, shortness of breath, chest pains (VS102-103), psychotic delusions (VS104), near death experiences (VS105) and the decompensation of chronic comorbidities (VS106-108) were related to a troublesome illness experience. The suddenness and unpredictability of changes in symptoms (VS109-113), the medial focus on severe COVID-19 (VS113-114) and excessive time spent in isolation (VS115) contributed to increased body alertness. Worries of relatives and the fear of not seeing them again impacted the illness experience during hospitalization (VS116-118). Some participants were afraid of or declined hospitalization (VS119-120).

Living Situation: Long isolation and extensions were burdensome (VS121-124). Available space, access to a garden, sunshine and distraction were protective against feelings of imprisonment (VS125-129). Parents described exhaustion due to family management, handling bad moods and social distancing while feeling sick (VS130-136), but also enjoying time together (VS127,137). Loneliness was often described as the worst aspect of COVID-19 (VS138-141). While many participants highlighted support from their social surroundings (VS142), the ban on visits led to a lack of care and support for people in need, e.g., unable to cook for themselves (VS143). Moreover, some patients had experienced stigma including from health staff (VS144-147).

Access to health care: Perceived health care deprivation during isolation was linked to unclear contact persons, busy hotlines, helplessness and anxiety (VS147-151). Health professionals among family and friends, pharmacists and complementary care providers and pulseoxymeters were important resources (VS152-155).

Personal strength and resilience: Having a health or scientific background helped (VS156), whereas previous trauma and mental health conditions impacted the illness experience and the handling of information overload from the media (VS157-159). A pragmatic or positive attitude and experience of handling stressful situations was considered important (VS160-161).

Feelings of guilt and worries: Several participants expressed constantly rethinking their contact behavior prior to knowing about their disease (VS162). Transmission led to feelings of guilt (VS163-165). Worries for infected family members also impacted the illness experience (VS164-166).

### 3.4. Triangulation of results

The key findings (*N* = 28) across all data sources can be found within the triangulation protocol (supplementary material S1). There was a high number of agreements between qualitative data sources (27 agreement, 1 partial agreement, 0 dissonance). Between the qualitative and quantitative data sources there was mostly silence (22 silence, 6 agreements, 0 dissonance), indicating that respondents often used the free space and notes to complement the information provided within survey questions.

## 4. Discussion

### 4.1. Summary of the main findings

To the best of our knowledge, this is the first large-scale mixed-methods study using surveys and interviews to explore the experience of people affected by COVID-19 within primary health care. Our results show that diagnostic barriers decreased after the initial adaptation of the health system to the pandemic; however, challenges in the area of contact management remained. Delayed diagnosis was related to worries and guilt of infecting others. The patients‘ illness experience was influenced by the course of the disease, the living situation and social surroundings during isolation, access to health care, personal resilience, spirituality and feelings of guilt and worries. A constant contact person within the health care system and coordinated information provision between different health care providers were identified as essential against feelings of health care deprivation and fear. Triangulation of results demonstrated a high agreement between key findings of the qualitative survey and interview data. Survey participants used the free space to complement these aspects relevant to their experience, whenever not covered by quantitative questions.

### 4.2. Comparison with existing literature

In many countries worldwide, including Germany, where this study was carried out, early diagnosis and isolation formed an essential pillar of the strategy for transmission control (39). This could explain the experienced improvements during the diagnostic process. Repeated testing and POC- antigen tests were confirmed as viable tools to reduce insecurities caused by long waiting times and initial false negative PCR results (40, 41). Our study, however, identified remaining challenges within the process of contact management, despite the massive training of containments scouts (without a necessary professional background in health) that served to encompass human resource constraints within Germany‘s public health system (39). Participants often perceived health authorities overburdened and uncoordinated, also due to the already described bureaucracy and lack of digitalization (42). Similar problems in coordination and communication have also been described in other countries (43, 44). COVID-19 diagnosis has previously been linked to rumination about prior contact behavior (45). Organizational problems during the process of diagnosis and contact management led to feelings of guilt and anger of having exposed others to an unnecessary risk and significantly impacted the illness experience.

In line with our study, narratives of patients in Prokop et al. (46) described common symptoms such as body- and headaches, as well as fatigue in a severity never experienced before. This unprecedented severity might be related to several pathophysiologic mechanisms, such as autoimmune responses, direct impairment of nerves or muscles or activation of nociceptive neurons (47). The unpredictable character of COVID-19 has been linked to a burdensome, undulating recovery process (48). In our study this unpredictability moreover led to an increased body alertness and fear, expecting symptoms to decline at any time. Comorbidities were confirmed as important modifiers of illness experience that should be carefully monitored (49). The mental burden caused by acute COVID-19 was furthermore increased by scaremongering media reports and one-sided political communication (50).

In agreement with hospital-based studies (16, 22), the physical environment (e.g., space and access to a garden) significantly influenced the illness experience during isolation. Loneliness has been described as a relevant theme during and after acute COVID-19, especially in older patients (51) and separation from children very difficult (52). We, however, found no studies identifying family management and conflicts, the bureaucratic burden linked to COVID-19 and organizing care for vulnerable family member outside the own household as additional stressors.

Continuity of care by GPs can lead to better general health outcomes (53). Studies from other countries moreover identified, that physicians at times experienced a closer relationship with their patients during the pandemic (43, 54). Our study identified a constant contact person within the health system as important pillar while experiencing COVID-19. Participants lacking a trusted contact person often described feeling left alone and “kafkaesk” while trying to get help and feared a worsening condition without anybody noticing. Apart from GPs, other care providers can play that role. Coordination between different stakeholders and clear guidance to patients should be assured to reduce stress caused by contradicting information (55).

In contrast to the possibilities highlighted in the literature (56–60), and the rapid expansion of telehealth in other countries (18, 20, 24, 61), surprisingly, video consultations were only reported by 1.5% of participants. In line with findings from Australia the telehealth tool mostly used by GPs in our study was the telephone (24). This could be explained by the lack of previous investments in further digitalization within the German Health Care system which hampered a rapid integration of other digital tools into GP practice (62, 63). The German health care system is currently developing strategies to expand home-based care in order to decrease the burden placed upon the hospital sector (64). Remote home monitoring models could reassure patients during home based isolation and care (19, 65), but more studies are needed to generate evidence for appropriate care (66) and the utility of different tools, such as pulseoxymetry (56, 66, 67). We showed that special attention should be placed on people living alone, with preexisting comorbidities or of old age. The same vulnerable groups have been identified in studies in the field of telehealth (18–20, 24, 65, 68). In line with our results, continuity of staff and a trusted relationship with the telehealth provider have been identified as important to patients (18).

### 4.3. Strengths and limitations

Our study results need to be interpreted considering some limitations: (1) The cross-sectional study design does not allow to gain sufficient data reflecting changes during the course of the pandemic. Moreover, emerging qualitative themes such as “loneliness” and “health care deprivation” were not assessed within quantitative variables to test for improvements after the first wave. They however kept emerging in qualitative interviews throughout the study. (2) A recall bias could play a role, particularly among participants who had experienced COVID-19 during the first wave. (3) Participants with a troublesome illness experience (due to either severe COVID-19 or other aspects influencing the experience) could have been more prone to participate and might be overrepresented. (4) Participating GPs were sampled by convenience and the results are therefore not representative. A selection bias of GPs more interested in patient-centered care and COVID-19 might also play a role and lead to an overestimation of the role in patient care played by individual GPs.

On the other hand, the recruitment through a large number of GPs and an adequate response rate compared to surveys in literature (69–71) allowed an inclusion of participants from all German states with a broad range of COVID-19 disease courses, comparable to the expected overall disease severity within the population (35). Although the lack of randomization does not allow representativeness, the geographic coverage, large data set and the methodological, data and investigator triangulation help to gain an idea of patients‘ experiences during acute COVID-19.

### 4.4. Implications for research and practice

Our study might contribute to a better understanding of home isolation as a new care setting, which will continue to play an important role in the context of the current and future health crises. The frequency of zoonotic disease outbreaks is likely to increase due to changes in climate and land-use, as well as livestock production and wildlife trade (72). While home isolation is a viable—and often preferred—option to hospitalization (18, 65), more studies are needed to generate evidence for appropriate care (66). Isolation should be as short as possible and not be prolonged without a strictly necessary reason (55). To provide and maintain patient care during isolation, the patient's condition, environment and responsibilities should be taken into account holistically. Patients of old age, with comorbidities or living alone should be followed up and support for basic needs must be assured. Isolated patients might also need support when in charge of care for other family members. Our study furthermore identified remaining challenges in the context of contact management, as well as coordination and information provision at the interface of GPs and health authorities that need to be addressed. Lastly, proactively signaling availability and clear guidance on what to do in case of worsening symptoms (52) (instead of waiting for patients to seek help) was an easy and very effective way of GPs to reduce the burden caused by acute COVID-19.

## Data availability statement

The raw data supporting the conclusions of this article will be made available by the authors, without undue reservation.

## Ethics statement

The study involving human participants was reviewed and approved by Ethical Committee of the University Hospital Würzburg (N.135/20 opinion dated 24th of June 2020). Survey participation was completely anonymous without any possibility to trace identities. The participants self-registered and provided their written informed consent to participate in qualitative interviews.

## Author contributions

SP, IG, and GR conceptualized the initial study and together with HS and AV, developed the study protocol and tools. SP, NL, LK, IG, AV, and GR were involved in study recruitment. NL and LK conducted qualitative interviews and transcribed the narratives. SP, GB, OM, and AF were involved in elaborating and carrying out the quantitative analysis. SP, NL, and LK conducted qualitative analysis and triangulation. IG supervised all processes. SP drafted the initial manuscript. All authors contributed during the process of reviewing and adapting the manuscript and approved the current version for submission.
