# Supplementary material for: Experiencing COVID-19, home isolation and primary health care: A mixed-methods study
Source: Front Public Health. 2023 Jan 10;10:1023431. doi: 10.3389/fpubh.2022.1023431 (PMC9872200; doi:10.3389/fpubh.2022.1023431)
Supplement: Supplementary file 1 [file Data_Sheet_1.docx]

Supplementary Material

# S1: Triangulation Protocol

**Triangulation protocol of main findings emerging from quantitative and qualitative results**

|  | Main Findings | Survey QUAN | Survey QUAL | Interview QUAL | Triangulation |
| --- | --- | --- | --- | --- | --- |
| **Experiences during the diagnostic process** | | | | | |
| 1 | Access to diagnosis improved during the course of the pandemic | Agree | Agree | Agree | Agreement |
| 2 | Waiting times for test results decreased | Agree | Agree | Agree | Agreement |
| 3 | Late diagnosis was linked to insecurities and ongoing transmission | No data | Agree | Agree | Silence |
| 4 | In the initial phase of the pandemic, participants often expressed understanding for the overburdened health system | No data | Agree | Agree | Silence |
| 5 | Some participants highlighted the dedication of individual GPs facilitating early access to diagnosis and test results, while others felt denied care | No data | Agree | Agree | Silence |
|  | | | | | |
| 6 | Patients often experienced an array of symptoms | Agree | Agree | Agree | Agreement |
| 7 | Participants reported unprecedented severity of common symptoms | No data | Agree | Agree | Silence |
| 8 | The duration of illness significantly impacted the illness experience | No data | Agree | Agree | Silence |
| 9 | The unpredictable course of disease let to an increased burden | No data | Agree | Agree | Silence |
| 10 | Preexisting comorbidities influenced the illness experience | No data | Agree | Agree | Silence |
| 11 | The focus of media communication on severe courses increased fear | No data | Agree | Agree | Silence |
| 12 | Extended isolation periods were troublesome | No data | Agree | Agree | Silence |
| 13 | Parents isolated with their children felt often overburdened, especially while being sick | No data | Agree | Agree | Silence |
| 14 | Organizing care for vulnerable family member was highlighted as significant problem during isolation | No data | Agree | Agree | Silence |
| 15 | Available space and access to a garden were important against feeling imprisoned | No data | Agree | Agree | Silence |
| 16 | Loneliness during isolation was linked to a burdensome illness experience | No data | Agree | Agree | Silence |
| 17 | Health care deprivation during isolation was reported | Agree | Agree | Agree | Agreement |
| 18 | Going through severe symptoms alone was related to fear of not getting help in time | No data | Partially | Agree | Silence |
| 19 | A personal contact person signaling accessibility made participants feel cared for and reduced anxiousness | No data | Agree | Agree | Silence |
| 20 | Most patients were cared for via phone | Agree | Agree | Agree | Agreement |
| 21 | In later stages of the pandemic more patients were attended personally within consultations | Agree | Agree | Agree | Agreement |
| 22 | Video consultations did not play a significant role | Agree | Agree | Agree | Agreement |
| 23 | Health authorities were perceived overburdened, especially at the beginning | No data | Agree | Agree | Silence |
| 24 | Organizational problems (incl. Corona Warn App) and the lack of digitalization (eg. Hand-filled contact lists) were raised as issue to halt transmission | No data | Agree | Agree | Silence |
| 25 | A lack of coordination among care providers and constantly changing rules led to diverging information and insecurities | No data | Agree | Agree | Silence |
| 26 | Fear and guilt of transmitting Sars_CoV2 significantly impacted the experience | No data | Agree | Agree | Silence |
| 27 | A pragmatic and positive view on life helped to handle the illness | No data | Agree | Agree | Silence |
| 28 | Religious faith as supporting pillar played a significant role in some participants | No data | Agree | Agree | Silence |

# S2: RKI Guidelines on Case Definitions used during the early phases of the pandemic

| Judgment based on clinical and epidemiological criteria | |
| --- | --- |
| Acute respiratory Symptoms of any severity  + Contact to a diagnosed case of COVID-19 within a maximum of 14 days prior to disease onset | Clinical or radiological suspect of a viral pneumonia without any alternative diagnosis  + No contact to a diagnosed case of COVID-19 |
| Clinical or radiological suspect of a viral pneumonia  + Link to a cluster of pneumonia within nursing homes or hospitals | Acute respiratory symptoms of any severity  + No contact to diagnosed case of COVID-19   - **But:** Work in the area of nursing, medical practice or hospital - *Or* being part of a high risk group - *Or* without having risk factors (COVID-19 diagnosis only in case of sufficient test resources) |

Suspected Case

Case in evaluation of differential diagnosis

# S3: English translation of Survey and semi-structured interview guide

**Date (please fill in the gaps): _ _-_ _- _ _ _ _**

1. When did your COVID-19 start?

*Please specify the approximate date and month (DDMM).*

**_ _ _ _**

2. Which of the following symptoms did you experience during COVID-19? (*Multiple answers possible)*

| ❑ | Fever: how high?______ | ❑ | Cough | ❑ | Sniffles |
| --- | --- | --- | --- | --- | --- |
| ❑ | Sore throat | ❑ | Headache | ❑ | Body aches |
| ❑ | Stomach ache | ❑ | Chest pain | ❑ | Shortness of breath |
| ❑ | Heart racing-/stumbling | ❑ | Alterations in smell perception | ❑ | Alterations in taste perception |
| ❑ | Diarrhea | ❑ | Vomiting | ❑ | Exhaustion |
| ❑ | Weight loss | ❑ | Skin rash | ❑ | Conjunctivitis |
| ❑ | Anxiety | ❑ | Disorientation | ❑ | Hallucinations |
| ❑ | Others, please specify: | | | | |

3. Have symptoms remained to this day?

❑ No ❑ Yes, please specify:_________________________________________________

4. How severe were your symptoms?

❑ No symptoms ❑ Symptoms of minor severity ❑ Symptoms of moderate severity ❑ Symptoms of high severity

5. Did you take the initiative to be tested for COVID-19?

❑ No ❑ Yes ❑ I do not know

6. For which reasons did you decide to get tested for COVID-19? (*Multiple answers possible)*

| ❑ | I experienced symptoms | ❑ | I was worried that I might be infected | ❑ | I was worried about relatives, who belong to a risk group |
| --- | --- | --- | --- | --- | --- |
| ❑ | As part of a scientific study | ❑ | For professional reasons (e.g. work in the health sector) | ❑ | I was asked to get tested |
| ❑ | To avoid prolongued quarantaine in case of a negative test result | ❑ | Others, please specify: |  | If someone asked you to get tested, who was it? |

7. How could you have been infected? (*Multiple answers possible)*

| ❑ | Unknown | ❑ | I had contact with a person with confirmed COVID-19 diagnosis | ❑ | I had contact with a person with suspected COVID-19 diagnosis |
| --- | --- | --- | --- | --- | --- |
| ❑ | I had stayed in a risk area in Germany | ❑ | I had stayed in a risk area outside of Germany | ❑ | Others, please specify |

1. Did you get tested for COVID-19 during the course of your disease?

❑ No ❑ Yes ❑ I do not know

*Please answer the following questions only, if you got tested for COVID-19 during the course of your disease. If you did not get tested or if you are not sure, whether you got tested, please* ***go to question 11****.*

2. For how many days have you been experiencing symptoms until you got tested for COVID-19? *Please write „0“ in case you did not have any symptoms at the time of your COVID-19-test.*

_ _ _ days

3. How long did the timespan between the start of your symptoms and your COVID-test appear to you?

❑ very short ❑ short ❑ appropriate ❑ a little to long ❑ much too long

4. Where did you get tested for the first time?

| ❑ | General practice | ❑ | Health department | ❑ | Emergency department |
| --- | --- | --- | --- | --- | --- |
| ❑ | Test site (e.g. university clinic) | ❑ | Company doctor | ❑ | Specialist practice (e.g. lung specialist) |
| ❑ | At home, by whom?_________ | ❑ | Others, please specify: |  |  |

5. Did you get tested multiple times?

❑ No ❑ Yes ❑ I do not know

6. If yes, how often?

______________

7. What was the test result? If you had multiple tests, please mark “positive” if at least one test was positive.

❑ positive ❑ negative ❑ I do not know (e.g. pending)

8. How long did it take until you were notified about your test result? *Please write down the number of days. If you were notified about your test results on the very same day, please write „0“.*

_ _ _ days

9. How did you experience the time span until you were notified about your test result?

❑ very short ❑ short ❑ appropriate ❑ a little too long ❑ much too long

10. Here you have space for further comments on your experience

Free text: ________________________________________________________________________________________________________________________________________________________________________________________________________________________________________________________________________________________________

11. If you did **not get tested** for COVID-19 **during your disease**, why did you not get tested?

Free text: ________________________________________________________________________________________________________________________________________________________________________________________________

12. Did you get tested for COVID-19 antibodies in the later course of your disease or after your disease?

❑ No ❑ Yes ❑ I do not know

16. If yes, what was the result of your COVID-19 antibody test?

❑ positive ❑ negative ❑ I do not know (e.g. pending)

1. How long did your disease last? *Please give the number of days.*

_ _ _ days

2. Did you receive sick leave during the course of your disease?

❑ No ❑ Yes If yes, how long? _ _ _ days

If yes, who issued your sick note? ____________________________________

3. Did you get diagnosed with a lung infection?

❑ No ❑ Yes ❑ I do not know

4. Were you hospitalized during the course of your disease?

❑ No ❑ Yes If yes, how much time did you spend at the hospital? _ _ _ days

5. Were you admitted to the intensive care unit?

❑ No ❑ Yes

6. Did you get ventilation?

❑ No ❑ Yes

7. If no, did you receive oxygen through your nose via a mask?

❑ No ❑ Yes

8. Which medication did you receive during COVID-19? *(Multiple answers possible)*

| ❑ | None | ❑ | Pain medication/ fever medication (e.g. Paracetamol) | ❑ | Antibiotics |
| --- | --- | --- | --- | --- | --- |
| ❑ | Antiviral medication | ❑ | Herbal remedies | ❑ | Homeopathic remedies |
| ❑ | Others, please specify: | | | | |

9. Which medication did you take by yourself (independently)? *(Multiple answers possible)*

| ❑ | None | ❑ | Pain medication/ fever medication | ❑ | Antibiotics |
| --- | --- | --- | --- | --- | --- |
| ❑ | Antiviral medication | ❑ | Herbal remedies | ❑ | Homeopathic remedies |
| ❑ | Others, please specify: | | | | |

10. Who took care of you during the course of your disease? *(Multiple answers possible)*

| ❑ | My family doctor | ❑ | Consultation hour for infectious diseases | ❑ | At an infectiologist’s office |
| --- | --- | --- | --- | --- | --- |
| ❑ | Specialist (e.g. lung specialist) | ❑ | Health department | ❑ | Others, please specify: |

11. How were you taken care of during your disease? *(Multiple answers possible)*

| ❑ | Primarily via telephone | ❑ | Personal consultation | ❑ | Via video consultation |
| --- | --- | --- | --- | --- | --- |
| ❑ | Others, please specify: | | | | |

1. Do you fear existential financial problems due to the COVID-19 pandemic?

❑ No ❑ Yes

2. Have you previously been diagnosed with mental illnesses? (Before the beginning of the COVID-19 pandemic)

❑ No ❑ Yes ❑ I do not know ❑ I do not want to answer

3. If yes, which? *(Multiple answers possible)*

| ❑ | Depression | ❑ | Anxiety disorder | ❑ | Addiction (e.g. alcohol, drugs, eating disorders) |
| --- | --- | --- | --- | --- | --- |
| ❑ | Post-traumatic stress disorder | ❑ | Adjustment disorder | ❑ | Others |

4. Did your illness experience scare, terrify or shock you so much, that you …

4.1 … suffered from nightmares within the last month or had to think of your disease even though you did not want to?

❑ No ❑ Yes

4.2 … tried very hard not to think about your disease, or tried very hard to avoid situations, which reminded you of your disease within the last month?

❑ No ❑ Yes

4.3 … were on guard all the time, or easy to frighten within the last month?

❑ No ❑ Yes

4.4. … felt numb or alienated from other people, activities or your environment within the last month?

❑ No ❑ Yes

**The following questions refer to your general state of health and to your state of mind**

1. How often did you feel impaired by the following symptoms within the last 2 weeks?

1.1 Little interest for or pleasure in your activities

❑ Not at all ❑ On single days ❑ On more than half of the days ❑ Almost every day

1.2 Dejection, melancholy or hopelessness

❑ Not at all ❑ On single days ❑ On more than half of the days ❑ Almost every day

1.3 Nervousness, fearfulness or tension

❑ Not at all ❑ On single days ❑ On more than half of the days ❑ Almost every day

1.4 Not being able to stop worrying or to control your worrying thoughts

❑ Not at all ❑ On single days ❑ On more than half of the days ❑ Almost every day

2. How old are you? *(Year of birth)*

_ _ _ _

3. What is your gender?

❑ male ❑ female ❑ diverse

4. In which state do you live? (main residence)

| ❑ | Baden-Württemberg | ❑ | Bavaria | ❑ | Berlin |
| --- | --- | --- | --- | --- | --- |
| ❑ | Brandenburg | ❑ | Bremen | ❑ | Hamburg |
| ❑ | Hesse | ❑ | Mecklenburg Western Pomerania | ❑ | Lower Saxony |
| ❑ | Northrine-Westphalia | ❑ | Rhineland Palatinate | ❑ | Saarland |
| ❑ | Saxony | ❑ | Saxony-Anhalt | ❑ | Schleswig Holstein |
| ❑ | Thuringia |  |  |  |  |

5. What is your highest education level?

| ❑ | Incomplete schooling | ❑ | Secondary Comprehensive | ❑ | Secondary |
| --- | --- | --- | --- | --- | --- |
| ❑ | Grammar | ❑ | Academic degree | ❑ |  |

6. How would you describe your employment status?

| ❑ | Employed full time | ❑ | Employed half time | ❑ | Self-employed |
| --- | --- | --- | --- | --- | --- |
| ❑ | Marginally employed | ❑ | Unemployed | ❑ | Not working (e.g. retirement, studies, parental leave) |

7. Do you work in patient care, at a doctor’s office or at the hospital?

❑ No ❑ Yes

8. Do other people live in your household, who are particularly vulnerable to a severe form of COVID-19?

❑ No ❑ Yes ❑ I do not know

9. Do children under the age of 18 live in your household?

❑ No ❑ Yes If yes, how many? _____________

10. Do you smoke? *(at least 1 cigarette/day or 5/week)*

❑ No ❑ Yes

11. Have you been diagnosed with the following illnesses? *(Multiple answers possible)*

| ❑ | COPD | ❑ | Asthma | ❑ | Cancer |
| --- | --- | --- | --- | --- | --- |
| ❑ | Other diseases of the lungs/ respiratory tract | ❑ | Pneumonia within the last 5 years | ❑ | Hypertension |
| ❑ | Other cardiovascular diseases (including stroke) | ❑ | Immunological diseases | ❑ | Diseases, which require immunosuppressant medication |
| ❑ | Obesity (BMI>40) | ❑ | Diabetes | ❑ | Liver disease |
| ❑ | Kidney disease | ❑ | Others |  |  |

13. What is your blood type?

❑ A ❑ B ❑ AB ❑ 0 ❑ I do not know

14. Did you receive vaccination against the flu in 2019?

❑ No ❑ Yes ❑ I do not know

15. In case you are 60 years or older: Did you receive the pneumococcal vaccine?

❑ No ❑ Yes ❑ I do not know

16. Here you have space for further comments:

**„Illness experiences of patients with COVID-19 in primary care– a mixed methods study“**

**Interview guide** Version 1 / August 6^th^ 2020

Interview-Nr.:

| Date of the interview: |  |
| --- | --- |
| Duration oft he interview: |  |
| Name Interviewer: |  |

**Introduction**

| Information provision, discussion of remaining questions:   - Welcome and introduction of the interviewer - Explanation of the interview procedure - Duration (about 20 minutes, but please take more time if there are more topics you would like to tell us) - Conditions of participation / data protection (according to participant information) - Brief explanation of the study objectives:   *During the anonymous questionnaire, we tried to get an overview of the course and the physical and psychological consequences of Covid19 in patients in primary care.*  *In this interview, we want to learn more about how you experienced the disease and its care. I am interested in your own experiences.*   - Any questions left? - Turn on voice recorder - Allocation/inclusion of the reference number |
| --- |

| Useful probes: What else comes to mind? / Can you tell us more about this?  Do you have another example here?  Could you please describe a typical situation?  What else do you mentally associate with this? |
| --- |

Interview- Questions

| I would now like to ask you questions about your experience.  Please take your time and tell us everything that comes to your mind. | | |
| --- | --- | --- |
| **Theme** | **Main Question** | **Further Questions** |
| **Illness experience** | Can you tell me about the time of your illness? | - How did you feel when you received the diagnosis? - What effects did the disease have? (on life, physically, mentally) - COPING: How did you deal with it? Was there anything that helped you? - Preventive behavior: How has the disease affected your behavior? |
| **Experience with health care** | How did you experience health care during the illness?  I am thinking, for example, of contacts with the practice, doctors, physician assistants or nurses, the health department, pharmacists or others. Please tell us what ever you can think of. | - What are your wishes regarding the care of Covid-19 patients?   (during the illness, after the illness) |
| Is there anything else on your part that you would like to add? Something that may not have been addressed until now? | | |

| **Post IDI Checklist** |  |
| --- | --- |
| Verification of the recording / Securely stored? |  |
| Cover sheet completely filled? |  |
| Socio demographic data complete? |  |
| Writing down important impressions/ Emerging Themes |  |

Sociodemographic characteristics of participants

| **Gender** | |
| --- | --- |
|  male  female  divers |  |
| **Age in years** | |
|  18-30  31-40  41-50  51-60  61-70  71-80  >80 |  |
| **Living situation** | |
|  rural (< 5.000 inhabitants)  Small town (5.000 - < 20.000 inhabitants)   medium size city (20.00-100.000 participants)  Big city (> 100.000 Einwohner) |  |
| **Have you been tested for Sars-CoV2?** | |
|  yes  no  I am not sure. |  |
| **Severity of COVID-19 course of disease** | |
|  mild  moderate (pneumonia)  severe (hospitalization)   critical (intensive care) |  |

Reflexions:

| Reflections on: How did the interview go, how did I feel? Did I feel like I was influencing the answers at certain points? Did I talk about myself? Were there any comprehension problems? Were there moments/causes for the flow of speech to stall? Please also insert other notes that may be relevant.       Further reflections:   Which research objectives/questions were addressed?    Are there topics that are relatively saturated?  Emerging themes:  Are there topics that should be further explored in other interviews?    Are there participants with certain characteristics who should be interviewed more? |
| --- |

# S4: Sociodemographic characteristics of qualitative interview participants


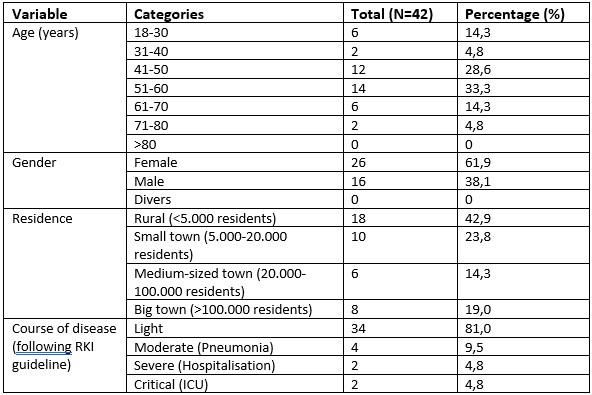


1. **S5: Qualitative results: English Translation of Verbatims**

| **Experiences during the diagnostic process** | |
| --- | --- |
| **SURVEY QUAL** | |
| **Accessing a test** | |
| VS1 | Everything was organized commendable. I received a test appointment with the family doctor immediately, 1 day later I got the result. It ran very smoothly. (PS 743) |
| VS2 | It was very difficult to get an appointment for the test. That was very nerve-wracking. (OS 35) |
| VS3 | Scheduling an appointment was far too tedious (over 100 calling attempts before getting through), I did not get any preference, despite being health care workers. (PS 47) |
| VS4 | I stood in a long queue with potentially infected people for two days. With fever and body aches. The organization was chaotic at that time (March). (PS18) |
| VS5 | According to the doctor, the fever was too low (38 °C) to be eligible for a test. (PS 144) |
| VS6 | Because no contact person could be specified. (PS97) |
| VS7 | While I was stuck to bed from March 22^nd^ until 29^th^ 2020 (I felt particularly bad at that time) and would have liked to be tested, nobody was interested and I wasn't allowed to be tested because I don't work in nursing and I'm not in professional contact with "many" people. (PS11) |
| VS8 | I was put on sick leave with the diagnosis “sinus infection”, although I lost my sense of smell from one moment to the other and I was sure to be positive (...). (OS 78) |
| VS9 | The test was rejected by the health department! Despite stating that my skier colleague had symptoms. (PS 100) |
| VS10 | The GP didn't want to test for a long time, he only tested me due to the pressure/instructions from the health department (PS 57) |
| VS11 | The replacement of my GP (which was on vacation) didn't want to have anything to do with Covid- 19 and let me down miserably. The statement was: "You do not have Covid-19". (PS 41) |
| VS12 | The mother was more seriously ill, so only she was tested (one household) (PS 156) |
| VS13 | My wife and I had the same symptoms so only my wife was tested, assuming if she is positive then so am I! (PS 147) |
| VS14 | "The tests need to be reserved for risk groups" was the answer from all clinics/doctors. (PS 144) |
| VS15 | I was unable to go to the testing station. (PS 669) |
| VS16 | Because I was feeling too bad and I couldn't leave the house. (PS 591) |
| VS17 | I went to the Corona emergency outpatient clinic without a referral. Since I had symptoms, the process was very unbureaucratic, the waiting time was short - I found it very positive! (PS 843) |
| VS18 | I am a hospital employee. I was symptomatic at the weekend, had a positive antigen rapid test on Monday, PCR smear taken, positive result received on Wed. (PS 985) |
| VS19 | I work at the university hospital (XX) and was able to have myself tested at a test center reserved for employees. (PS 912) |
| VS20 | My GP informed me about this (positive test result) on a Sunday. (PS 193) |
| VS21 | The GP even contacted us outside of office hours. We were also immediately asked if other people had symptoms and we were informed about the quarantine. (PS 195) |
| **Waiting for test results** | |
| VS22 | Feeling tense, hopefully not, what if... (PS 325) |
| VS23 | The time between the test and the result was very long. That was emotionally very exhausting. (PS 490) |
| VS24 | The uncertainty causes depression (because other people living in the household are also affected) (PS 89) |
| VS25 | It took 4 days from the time of the first complaints to the test result, in the meanwhile this meant uncertainty for me, my roommates and contact persons. If they had been infected, they could have infected many other people. (PS 259) |
| VS26 | Waiting time was too long > problems with employer > quarantine of employees needed to be decided (PS 5) |
| VS27 | They said I only had to wait 24 hours (I'm a kinder garden teacher and had no complaints, so I had to work until receiving the result). More than 3 days are simply too long in these cases. (PS 255) |
| VS28 | The test took place far too late, only being in contact with a person who tested positive. By then your whole family had been infected. (PS 84) |
| VS29 | Theoretically, I could have walked around and worked for 4 days before my partner received his positive result (4 days after taking the sample). (...) Where do the high numbers come from? Good question! (PS 828) |
| VS30 | Since the rapid test was positive, I could prepare myself for the pending result of the PCR test. (PS 285) |
| VS31 | Our GP carries out quick tests. The result was clear within 2 minutes. It was confirmed by the laboratory the next day. (OS 30) |
| VS32 | 1^st^ test: PCR test negative in the clinic on November 11^th^. 2^nd^ test: rapid test positive at the GPs on November 16^th^. Since the rapid test was immediately positive > I immediately informed all contact persons and went into quarantine. (PS 918) |
| **Interviews QUAL** | |
| **Accessing diagnosis** | |
| VS33 | Then I, uh, yes, I, uh, did a test in (another big city) at the airport, because, uh, the test centers in (state) were still somewhat unstructured. (15 NL) |
| VS34 | First of all, it took a crazy long time until I somehow got someone on the line, (...) then I was told, that they don't give appointments like that, that I would have to go to the family doctor the next day. And the family doctor was - of course - on autumn vacation. Then I had to get an appointment with another GP. (12NL) |
| VS35 | It happened on the weekend, of course on a Saturday, so we called the medical emergency service and asked what to do “Do you have any fever?” - but I didn't have that incredibly high fever, just about 38 degrees, and then they said “no”, that they wouldn't test, that I should go to the GP on Monday. (9NL) |
| VS36 | The symptoms were clear. Well, I'm a microbiologist, I know something about that subject, so everything was clear to me. (...) one falls through this grid! So that was, that wasn't a well-established plan or instructions, eh, guidelines, you just fell through that grid. (4 LK) |
| VS37 | He immediately built up a tent, a tent in front of the practice, in the garden. So you didn't have to go into the practice. And that actually went smoothly. (2 LK) |
| VS38 | And then I was supposed to take this smear myself, through the window, everything was secure and so on. That was really well prepared. (3 LK) |
| VS39 | I thought it was very, very, great of my GP to call me near 10 o clock in the evening, just to inform me about the test result and to give me a short list of to do`s. (14 LK) |
| VS40 | The next day the doctor called me and said, well, now, erm, she also had information that it might have something to do with it (COVID-19). The fact that you can't smell or taste anything. I should come by and get tested. But by then ten days had already passed. (6 LK) |
| VS41 | As a family doctor, I can't say “We don't do Corona”! That's not possible. But that's how this practice handled it. (11 LK) |
| VS42 | Well, and then the usual alarm. You had to inform the health department, get in touch, inform employers, and it takes you a day or two to organize everything and, yes, to get over the initial shock. (11 LK) |
| VS43 | What felt like hundreds of millions of calls to finally manage to get the on-call service to come and test the guys on the doorstep on Thursday at eleven o'clock in the night. (...) And, well, all positive. And then, suddenly, my one son's class was completely quarantined. (...) Because nobody was able to test our children and we weren't capable of going anywhere. (22 LK) |
| VS44 | I experienced it as very difficult, that none of my contacts were tested at all (...) For example my boyfriend, he was with his family and was not tested at first (...) he then had to stay in quarantine for a whole week longer. That was totally chaotic. (2 NL) |
| VS45 | Then she said that someone from the health department would call and take a smear, but that was it, there was no response for several days, they were overburdened at first. (2 LK) |
| VS46 | Well our health department was completely overwhelmed. (...) then she says that I should send her the list with the contacts, but that they wouldn`t call anyone anyway. Because they didn't have the time to do it. (6 LK) |
| VS47 | And then, I also found out that the information had not been passed on (within the health department). And always a different contact person (...) I had to answer the same thing on several days in a row. (4 LK) |
| VS48 | And that's when I lost a bit of confidence during the phone calls with the health department. Ehm, because you simply have three contacts persons in charge and get three different statements. (14 LK) |
| VS49 | And this absurd list of contacts that you have to fill out by hand and send back in the mail. I thought, “yes, hm, then I'm not surprised that the contact tracing doesn't work”. (23 LK) |
| **Emotional response** | |
| VS50 | And actually, when it was on television or on the news, that there actually happens a lot in private, I could not find an explanation. I could not believe it. Then it hit myself. (7 NL) |
| VS51 | Then we said at first, hm, Corona, no. That’s not possible, we are all fit and healthy (…) I do not have something like that, I do not have asthma, nothing, I am normal, healthy as a horse. (19 NL) |
| VS52 | Until then I did not at all think that – erm – that something might not be alright. It was really like a slap in the face. (1 NL) |
| VS53 | When we were notified, we were at first just sitting there, and at times you do not even know: Yes. And now, how will things go on now? Well, and, well in the evening when we got notified, that was just horrible for us, I have to say. (17 LK) |
| VS54 | It did not really crush me or something like that. Certainly one has also already seen all those pictures on television and of the intensive care unit and so on, but I can tell you, I was not totally frightened or so. I just lived with it somehow and I just thought, well, let’s see how things will develop. (7 LK) |
| VS55 | The certainty – well that has helped me extremely, that is now what it is. One cannot do anything. (23 LK) |
| VS56 | Then about the mental History: After, erm, the test was positive, then you – of course – start to think, what was going on back than at the time – erm – when one could have passed it on. (24 LK) |
| VS57 | I had the positive test result. Where I of course thought, hmm, now you have pretty much been infectious for five days and you were walking around! (23 LK) |
| VS58 | Yes. I sort of lost the ground under my feet, because, well I had asumed up until this moment that it was certainly nothing, but … ist was then just rather the, yes, psychological burden where I thought, my god, now I was there at work, I was here, I was here, I was there. (6 LK) |
| **Illness experience** | |
| **SURVEY QUAL** | |
| **Disease progression and symptoms** | |
| VS59 | I would normally not have gone tot he doctor because oft he symptoms! (PS 100) |
| VS60 | My COVID-19 disease was form e the most severe illness. Ihad never experienced fear of death. Even turning around in the bed was a real feat. (OS 68) |
| VS61 | I experienced the illness and its course worse than the chemotherapy I received in 2012. (PS 514) |
| VS62 | During the time at the hospital I was so sick, that I thought I had to die. For 10 days I had a fever of 40 degrees, even though I took 8 tablets of paracetamol for at least 7 days. Eight … After the hospital stay I suffered from sleep problems for weeks. (PS 12) |
| VS63 | In the beginning the headache and body aches were very severe. I did not know, that a body could hurt like that. No continuous recovery … one day was better, after that again worse, back and forth, after 1 week cough and altogether very weak! (PS 278) |
| VS64 | The headache was very, very severe. I had to fight a migraine, but this headache was 10 times worse. The complete loss of taste and smell sensation was very frightening. The body aches and this weakness were extremely severe. (PS 265) |
| VS65 | At the middle of the illness I had about 3 to 4 days in which I suffered from an extremely severe cough and sometimes I had to vomit due to physical exertion. (OS 2) |
| VS66 | My main problem was an incredible fatigue. I could only move step by step, like a 95-year old man. (PS 191) |
| VS67 | Enormous exhaustion never experienced before. (...) It took relatively long (over two weeks) to feel able again to go out, or to get the body and circulation going again. (OS 65) |
| VS68 | I experienced the acute phase of the disease as very stressful, due to the many symptoms and the fact that new symptoms were added continuously. (PS 939) |
| VS69 | Changes in symptoms, e.g. feeling relatively well > sudden fatigue. (PS 779) |
| The Mental Burden caused by COVID-19 | |
| VS70 | Increasing complaints, that`s what made me incredibly afraid: "what's to come?", this led to an inner cinema > “intensive care unit?” (PS 220) |
| VS71 | Panic attacks associated with nocturnal palpitations; Fear of late sequelae. (PS 775) |
| VS72 | There was always fear. (PS 238) |
| VS73 | The fear of a bad outcome stoked by the media and politics was also burdensome. (PS 635) |
| VS74 | I belong to a risk group because of pre-existing conditions and age, my husband as well, so we worried a lot! (PS 508) |
| VS75 | The two weeks quarantine in the small 2 room apartment without garden and balcony on the first floor were hell. 14 days without meeting anyone was not nice, and I do not wish it to anyone. All alone in the apartment, while my mother was isolated with COVID-19 in another apartment, also alone. (PS 868) |
| VS76 | I felt alone, because I live alone! I have a 90-year-old mother who I had infected, I felt completely overwhelmed. I had to stay alone in the hospital for 4 weeks. This was a terrible experience, no visits allowed, just terrible!!! It was impossible to organize help for my mother, so in the end I just called the emergency service out of necessity. (PS 566) |
| VS77 | The fact to be alone with the complaints, being in quarantine, is stressful and difficult to bear. (PS 939) |
| **Access to health care** | |
| VS78 | I was never examined after the initial test (e.g. for pneumonia). (PS 363) |
| VS79 | Personally, I have experienced the disease as very difficult, but the worst thing is that you are not allowed to go to see a doctor, “in case of shortness of breath, go to the hospital” was the advice. (PS 303) |
| VS80 | The GA did not provide any information at any time! GA not reachable by phone. I “flew” out of the waiting loop umpteen times, after 15 min! (PS 658) |
| VS81 | I felt left alone with Covid and in quarantine. (OS 64) |
| VS82 | I felt left alone as there was no contact person in case of worsening symptom or to answer general questions. (PS 104) |
| VS83 | I felt left very alone in March. There was no one to examine you. No one helped you. You were locked up and you had to see how to cope with it. I was happy that my 21 year old daughter (nurse) took such good care of me. Since I am a single parent with 3 children - one of them being a high-risk child – the experience was absolutely no gift. (PS 84) |
| VS84 | During the illness, there was no support, neither from the health department nor from the family doctor. The only thing that you could do was to request a prescription for cough from the GP by phone. Unfortunately, no consultations took place. I was very helpless during that time. No examination for pneumonia took place. (PS 62) |
| VS85 | No care during the illness phase (nor afterwards). Insecurity. (PS 102) |
| VS86 | The perplexity when calling the 116117 after the first symptoms appeared in the evening was frightening. (PS 1001) |
| VS87 | The doctors gave the impression of ignorance due to lack of experience or too little continuing education (OS 45) |
| VS88 | There was no care. The family doctor referred to the health office, but they only called after the quarantine had already ended, as my positive result probably did not reach the office. (OS 88) |
| VS89 | I didn't feel cared for well. The health office and family doctor called irregularly and passed on diverging information (PS 616) |
| VS90 | Was looked after by 2 health departments (XX1 because of job, XX2 because of place of residence). Each health department gave different information regarding new tests, start of work, quarantine, etc. It was all very confusing. (PS 541) |
| VS91 | The health department was very disorganized. They had no computer support and did everything manually. This led to multiple calls from different medical officers who asked the same thing... It was all chaotic! We had to beg to run tests, because we had no symptoms. (PS 610) |
| VS92 | The care provided by the health department during quarantine was great. (PS 72) |
| VS93 | There was no need for care. (PS 657) |
| VS94 | The daily telephone contact with my family doctor was a great mental support!!!! (PS 659) |
| VS95 | During this time, there were many ups and downs. And thanks to my family, friends and my great family doctor, I survived this not so easy time. (PS 494) |
| **Interviews QUAL** | |
| **Disease course and progression** | |
| VS96 | I really never had such a stabbing headache before in my life. (3 LK) |
| VS97 | And then I just got really massive problems with my muscles, I mean with muscle breakdown. Such pains that I really thought: No, I can´t do this anymore. It´s just not possible, I am not able to endure this. (20 LK) |
| VS98 | This dullness (...) feeling “I am actually thirsty, but to get up, to get something to drink is just much too much effort”. (23 LK) |
| VS99 | These three weeks were so terrible! (...) we didn't watch TV, we didn't listen to the radio, we didn't answer a phone, I didn't write Whatsapp. Nothing, zero. We just lay here and slept (...) and looked out of the window. (6 NL) |
| VS100 | High fever, palpitations, cough (...) I just somehow caught everything. Gastrointestinal problems, pain, exhaustion, everything. (...) I was in quarantine for 17 days and these acute symptoms lasted for, I´ll say, three weeks. (8 NL) |
| VS101 | Then I got fever, 39 degrees Celsius lasting for over 10 days. Then very bad nerve pain, trigeminal pain, uh, pain at the nerve endsdon the back, on the arms, (...) that was very bad. Then, gastrointestinal symptoms, exhaustion, uh, nausea, shortness of breath, I couldn't walk up stairs without having to sit down or lie down again. Tiredness too. Whereever I sat down, I fell asleep. (10 NL) |
| VS102 | I always had the feeling that 2 or 3 kg of weight were pressing on my chest. (...). I had to make a real effort to be able to breathe in. And then at night I somehow caught myself once or twice, how I came up, woke up and gasped for air (...). After this I couldn't sleep anymore, because I was afraid to somehow suffocate or whatever, to not wake up again. (7 NL) |
| VS103 | I just woke up and realized that it was harder to get air. But I wouldn´t speak of dyspnea, but rather of shortness of breath - you have the feeling that you can no longer breathe in deeply. It is kind of... (breathes in demonstratively for a moment), and then there is a barrier, yes? And, um, then you really panic! Because you always hear these other things, so to speak, and think, “man, I thought that I was over the hump, but - probably not.” (11 NL) |
| VS104 | Insane hallucinations and the like. (...) I've seen all sorts of things, during the day. And every nurse who entered my room, or even the doctors; I always had the impression that they wanted to kill me. (16 LK) |
| VS105 | There was such a, yes, a divine rest. I didn't even feel myself. So I didn't see anything, nothing, it was just a feeling where I felt really safe. And then I thought to myself, “well, this is death now”. And actually, you can't die more beautifully. If people knew how great it is, that you don't have to be afraid, and you don't feel any pain! (16 LK) |
| VS106 | It was such a period of time where I realized that I was ... yes, closer to my threshold again. Moving along my stimulus threshold, meaning that it may lead (…) to a small episode. (11 LK) |
| VS107 | I also have Parkinson's. And during the, yes, during the virus, when I also had a fever, my movements were quite slow. So that kind of troubled me. Despite my medication. (10 LK) |
| VS108 | Then my blood pressure crushed, because I continued to take my medication. So I found myself lying on my tiled floor in the bathroom (...) And when I gained consciousness I had three teeth broken out. (4 NL) |
| VS109 | I fell unconscious and fell over twice during the night when I tried to get up. (...) What amazed me was the speed with which the symptoms then spread to me. (19 LK) |
| VS110 | Moreover, there was this rapid, rapid, eh, worsening of the situation. (...) I have to say that there were also these very, very strange symptoms. I had pain in the chest, in the area of the heart, I had pain in the lungs. (4 LK) |
| VS111 | So, among the 6 people there were also 6 different symptoms.( 22 LK) |
| VS112 | Constant up and downs. It wasn't like a normal cold. I had phases in between, where I was fine, where I thought, “oh, I'd rather go to work now”, and the next day I suddenly felt worse again. That was, how I felt, I perceived them as flare-ups. (16 NL) |
| VS113 | This uncertainty – “How will my disease course be?” Can you predict it on a day to day base or are there no clues at all? And then, of course, you stupidly read a lot, also a lot on the Internet. And of course there are all kinds of thigs there: People that got bad on the second day, but also people that only went really bad after three weeks. So, of course that doesn`t help. (1 NL) |
| VS114 | To always have in the back of your head: “that can end very badly”, is not - is not beneficial. And I don't think it's okay for the state to communicate it that way, because the first thing you hear on the radio is how many have died of Corona. (11 NL) |
| VS115 | If you have so much time, and also have so much time to think, you are somehow much more sensitive to your body, and you experience it much, um, more precise, and you, you pay much more attention to it. Things that I might not have noticed in everyday life. (1 NL) |
| VS116 | That`s what really troubles me: that they had to go through it. More than my own experience, because I am still here! (...) my husband was really, really battered. (16 LK) |
| VS117 | That was really bitter for my wife that she thought, she thought… she would never see me anymore. (19 LK) |
| VS118 | I remember the last thing I texted to my wife, that was still possible at that moment, on the day I was admitted. The last night I wrote to her that I love her and, about everything, and think about her, and that - these are very, very deep emotions. (19 LK) |
| VS119 | The doctor said, the family doctor: “if that doesn't improve now, we have to consider hospital admission”. And I already knew that it usually doesn't end, well when you're ventilated. Therefore, I just wanted to avoid this, for heaven's sake. (15 NL) |
| VS120 | The doctors also recommended us to go to the hospital, uh, because of our pneumonia, uh, which we both had (...) we both thought, “No. Catching additional germs there in the hospital, uh, won´t help.” |
| **Living situation during isolation** | |
| VS121 | If I ever catch that again, I`m mostly scared of another isolation. (15 LK) |
| VS122 | so it was already bitter. In the end, I was in quarantine for almost five weeks. (22 LK) |
| VS123 | And then there was frustration, above all because the result took so long. And, and then, because the quarantine was then also postponed, and, yes, well, that was no fun, (...) that was also stressful for me, somehow. (21 LK) |
| VS124 | And then it wasn't even clear, “will my symptoms be really gone 48 hours before my quarantine ends? Will I be free then?” (...) I had the constant thought in my head: “if I have to stay locked in for another week - 28 days of quarantine, no! No!” (13 LK) |
| VS125 | Well, I'm lucky that I have a house and a big garden, and that my children could always go to the garden to play and so on, well, we're are really fortunate in that way. (17 NL) |
| VS126 | Well, we have a big garden, the weather was nice, so I had no problems with it (isolation) at all. 5 |
| VS127 | I could at least go out into the garden. Briefly, with my daughter, there was a good moment on a Sunday when I thought things were going better. We played a little soccer. This was a real feel-good moment. Much better than being locked up the whole time. (9 LK) |
| VS128 | I felt... splendid. I used the time to renovate half of my house. (11 LK) |
| VS129 | We had something to do. That was the main thing. I think if we had had nothing to do, it would have been more dramatic. (15 LK) |
| VS130 | there are moments, I have to say honestly, where I thought I should have gone to the hospital. Just that the family perceives, “hello, I'm not feeling well.” (...) You can't take a break. Not if you have so many children. (22 LK) |
| VS131 | Then you have to constantly pass on the names, six times (referring to one phone call for each of the six household members). And the date of birth, six times. Then the quarantine. Everyone had different quarantine periods. (...). And all that during the week when I was feeling really bad. (22 LK) |
| VS132 | My child was half a year old and as a mother you just have to function (laughs), but somehow it was quite exhausting, I had quite a lot of body aches. (1 LK) |
| VS133 | Three weeks, where we were not allowed out. That was extremely exhausting. Because we were locked in together, all four in a tight space. You can't avoid each other much, and at some point the ceiling seems to crush on your head. The children at some point, get, eh, really dizzy and the potential for confrontations increases. (18 LK) |
| VS134 | And then the family, and also the hormones and puberty: You had to bring had to reconcile everything, that's - well, it was really a very stupid time. (22 LK) |
| VS135 | Well, I had to handle the bad mood of my husband. (1 LK) |
| VS136 | The separation from the daughter, that was really hard. She was really at times sitting in front of the door and cried and could just not come in. (17 LK) |
| VS137 | Luckily, our family fits very well together. With my husband, too. We went through that allright. The children too. Retrospectively we keep on saying this: No matter how hard it was, they all participated very well and it worked very well. (17 LK) |
| VS138 | I think this isolation is one of the worst things about the disease itself. Not even really the medical aspects, but actually, that you are just so alone (1 NL) |
| VS139 | And then my mom on the phone, who was also uh, completely depressed and cried constantly, and, and, we couldn't either. What could I do? We didn't see each other at Christmas, we didn't see each other on New Year's Eve. (6 NL) |
| VS140 | That was not really easy for me, I mean psychologically, because I was completely alone for 10 days, in this granny flat. (2 NL) |
| VS141 | Then, eh, the grandchildren, no one came, everyone kept the distance. I have 10 grandchildren (laughs), yes. And all just by far. And, me, that was... (8 LK) |
| VS142 | the family and neighborly network, who then also offered themselves to go shopping and so on. So that was good for you, that you knew you weren't hanging in there alone. (5 LK) |
| VS143 | that's the bad thing when you're in quarantine: no one can help you. They, that is, our friends provided us with food, (...) but I was not able to cook anything for three weeks. (6 NL) |
| VS144 | You are avoided a bit. Because people didn´t know how to handle the situation. “What`s that new thing? They have Corona, we have to keep on walking, quickly!” Nobody said anything, but at the beginning you did notice that some people didn´t want any contact. (9 LK) |
| VS145 | At the end of October, I was the first case among our closer circle of friends and relatives (...). You fell a little, well I´ll use the word “leprous”, but maybe that`s a little extreme. But you were avoided and asked questions, people were interested and so on. (21 LK) |
| VS146 | Then the fact that I was at hospital. When the paramedics dropped me off there, while leaving the ambulance they said: “Now we can disinfect our ambulance for hours, again.” that actually affected me more than anything else. (16 LK) |
| **Access to health care** | |
| VS147 | And also on the Internet. Everywhere, where I had informed myself it always said: Call this hotline. The hotline and the health office. But they were both unreachable. (4 LK) |
| VS148 | At some point you get to a point where you`re just scared. Who will help me? It was just impossible to get help (...) I didn't know how bad it would get the next night. “Will you still be able to get up, talk on the phone, or do anything?” (...) You can tell it's getting worse and there's no help. (4 LK) |
| VS149 | This helplessness is evil. This is madness. And above all, our friends also said to us: “what would have happened, if you wouldn´t have had anyone? And you would have been here, you would have passed out in here, no one would have even noticed!” (...) So that, that's, that's terrible! And that's what, uh, has also affected me a lot, psychologically. (6 NL) |
| VS150 | If you have to go through all this on your own, you just feel so alone and abandoned. Well, I really felt like that a little bit. Because, of course, no one looks at you. Yes? And the only option that you have is to go to the clinic. So, I was very scared during the illness. (8 NL) |
| VS151 | We were already very helpless here at home. I really have to say that. (7 LK) |
| VS152 | My colleague somehow took a blood sample and investigated to see if I needed heparin or something, and my oxygen didn't go below 93. So somehow - my daughter gave me infusions, she is a medical assistant - yes, well. That means I could really handle it well at home, which is of course the exception. (13 NL) |
| VS153 | At the pharmacy – she also told me with what to rub my chest, what can help against the cough. Well, I really felt in good hands. (23 LK) |
| VS154 | but I feel good within the so-called alternative, uh, with osteopathy, I feel quite well cared for (24 LK) |
| VS155 | What was also good: I have a pulseoxymeter here at home, where we could measure SpO2 and the pulse rate here at home. We were able to control for ourselves whether there are problems that we do not see at first glance. (14 LK) |
| **Personal strength and resilience** | |
| VS156 | I also studied physiotherapy, which means that I know about- I am already familiar with science. (14 LK) |
| VS157 | Five years ago, my husband died next to me in the car while driving, of a heart attack. (...) that influenced me a lot. I felt so anxious when he constantly got up, went to the window, gasped for air. I felt, I felt anxiety all the time. (...) I was so afraid that this would happen to me again, to lose a beloved partner. (6 NL) |
| VS158 | What also plays a role in my context is that I am in psychological treatment since 2007, due to a severe burnout. Therefore, this now affected me a lot, psychologically. (6 NL) |
| VS159 | Although the symptoms were not so dramatic or actually not dramatic at all, I obviously consumed the news. 20 hours a day! (...) these trucks in Spain, or, or Italy, where they removed the bodies. (...) Then came these messages: "Ahh, there may be symptoms later", you somehow become so noisy and hear the grass growing, and ... (sighs) (11 LK) |
| VS160 | I am very pragmatic in such situations. (...) I thought to myself, “it's not going to be that bad. Now you just wait in quarantine, and if it gets worse, you just go to the hospital.”(16 LK) |
| VS161 | I'm quite solid in, in self-confidence, in appearance and stuff like that. Also due to my job as a team leader. (...) If you are in charge of 30 people, eh, you can tolerate a bit of pressure. (...) you are not quite so unstable. If such a thing suddenly happens to you, you don´t get that shocked and don´t worry too much. (18 LK) |
| **Feelings of guilt and worries** | |
| VS162 | After getting the positive results, your thoughts start to turn and turn: “Man, with whom, where, eh, what could happen, did someone else get sick?”. That was actually the most stressful situation for me, psychologically. (24 LK) |
| VS163 | I had feelings of guilt, or simply blamed myself, yes, that I may have infected people. Even in my family (...) “hopefully nothing will happen, with my parents who are already over seventy years old.” (...) Since I live in a patchwork family, it naturally also hit the father of my children. And he actually really blamed me, so (laughs) that was somehow, so, somehow a bit stressful. (6 LK) |
| VS164 | She still has complaints breathing, until today. It hit her even worse than me. (...) Yes, there was also a lot, a lot of guilt -guilt (laughs), I felt, very guilty. (8 LK) |
| VS165 | Of course, this burdens me, because I am really worried, worried, yes, because he actually got it through me (...) and I of course wish that everything goes away as soon as possible, eh, and that he then, eh, gets normal and fit again. (24 LK) |
| VS166 | I then, uh, also had a lot problems sleeping - well, uh, my partner, who had it so, so, bad in the lungs, he had 83% of oxygen saturation, didn´t go to hospital. He got up every night, to the window, so that I hardly slept for the first three weeks, and of course, that affected me a lot. (6 NL) |

Identifier: OS= Online Survey Number X; PS= Paper Survey Number X; NL/LK: Interview number X conducted by NL/LK

1. **S6: Qualitative results: Original German Verbatims**

| **Experiences during the diagnostic process** | |
| --- | --- |
| **SURVEY QUAL** | |
| **Accessing a test** | |
| VS1 | Lief alles vorbildlich. Sofort Testtermin beim Hausarzt erhalten, 1 Tag später Ergebnis. Lief sehr reibungslos. (PS 743) |
| VS2 | Es war sehr schwierig einen Termin für die Testung zu bekommen, was sehr Nervenaufreibend war. (OS 35) |
| VS3 | Die Terminvereinbarung war viel zu mühselig (über 100 Anrufversuche bis Durchkommen), keine Bevorzugung trotz Mitarbeiter in Gesundheitsberuf (PS 47) |
| VS4 | Habe zwei Tage in der langen Warteschlange mit potentiell Infizierten gestanden. Mit Fieber und Gliederschmerzen. Damals (März) chaotische Organisation. (PS18) |
| VS5 | Fieber war laut Arzt mit 38 Grad zu niedrig um Anspruch auf Test zu haben (PS 144) |
| VS6 | Weil keine Kontaktperson angegeben werden konnte. (PS97) |
| VS7 | Als ich von 22.03. bis 29.03.2020 (da ging es mir besonders schlecht) völlig daniederlag und gerne getestet worden wäre, hat das keinen interessiert und ich durfte nicht getestet werden, weil ich nicht im Pflegedienst arbeite und nicht mit "vielen" Personen beruflich in Kontakt bin. (PS11) |
| VS8 | Ich würde mit einer Nebenhöhlenentzündung krankgeschrieben, obwohl ich von einem Moment zum anderen den Geruch verloren habe und ich sicher war, positiv zu sein (…). (OS 78) |
| VS9 | Test wurde vom Gesundheitsamt abgelehnt! Trotz Angabe, dass Skifahrer-Kollege Symptome hatte. (PS 100) |
| VS10 | Hausarzt wollte lange nicht testen, erst auf Druck/Anweisung des Gesundheitsamtes (PS 57) |
| VS11 | Die Vertretung meines Hausarztes (war im Urlaub) wollte wohl mit Covid 19 nichts zu tun haben und hat mich jämmerlich im Stich gelassen. Aussage: "Sie haben kein Covid 19". (PS 41) |
| VS12 | Mutter schwerwiegender erkrankt, somit nur sie getestet (ein Haushalt). (PS 156) |
| VS13 | Meine Frau und ich hatten dieselben Beschwerden, deshalb wurde nur meine Frau getestet, man ging davon aus, wenn sie positiv ist dann bin ich es auch! (PS 147) |
| VS14 | "Die Tests seien für Risikogruppen aufzuheben" war die Antwort aller Kliniken/Ärzte. (PS 144) |
| VS15 | Ich war nicht in der Lage die Teststation aufzusuchen. (PS 669) |
| VS16 | Weil es mir zu schlecht ging und ich das Haus nicht mehr verlassen konnte. (PS 591) |
| VS17 | Ich bin ohne Überweisung in die Corona-Notfall-Ambulanz gegangen. Da ich Symptome hatte, war der Prozess sehr unbürokratisch, die Wartezeit kurz - habe es sehr positiv empfunden! (PS 843) |
| VS18 | Ich bin Krankenhausangestellter. Am Wochenende symptomatisch, am Mo Antigen-Schnelltest positiv, PCR-Abstrich abgenommen, am Mi positives Ergebnis erhalten. (PS 985) |
| VS19 | Ich arbeite am UK XX und konnte mich dort an einer Testzentrale extra für Mitarbeiter testen lassen. (PS 912) |
| VS20 | Mein Hausarzt hat mich am Sonntag darüber informiert. (PS 193) |
| VS21 | Der Hausarzt hat uns sogar außerhalb der Sprechstunde selbst kontaktiert. Auch wurde sofort gefragt ob weitere Personen Symptome haben und uns aufgeklärt über die Quarantäne. (PS 195) |
| **Waiting for test results** | |
| VS22 | Angespannt, hoffentlich nicht, was wäre wenn... (PS 325) |
| VS23 | Die Dauer zwischen Test und Ergebnis war sehr lange. Das war emotional sehr anstrengend. (PS 490) |
| VS24 | Die Ungewissheit macht depressiv (da andere Personen die mit im Haushalt leben ebenfalls betroffen sind) (PS 89) |
| VS25 | Vom Zeitpunkt der ersten Beschwerden bis zum Testergebnis waren es 4 Tage, dazwischen herrschte Ungewissheit bei mir, meinen Mitbewohnern und Kontaktpersonen. Hätten sich diese angesteckt, hätten sie sehr viele weitere Menschen anstecken können. (PS 259) |
| VS26 | Zu lange Wartezeit > Probleme mit Arbeitgeber > Quarantäne der Mitarbeiter stand infrage (PS 5) |
| VS27 | Es hieß ich müsste nur 24 Stunden warten (bin Erzieherin, keine Beschwerden, musste also arbeiten bis das Ergebnis da war), da sind über 3 Tage einfach zu lange (PS 255) |
| VS28 | Der Test fand viel zu spät statt, erst nachdem man mit einer positiv getesteten Person in Kontakt war. Da hatte man dann schon seine ganze Familie angesteckt. (PS 84) |
| VS29 | Theoretisch hätte ich bis zum positiven Ergebnis meines Partners (nach 4 Tagen nach Probenahme) also 4 Tage rumlaufen und arbeiten können. (…) Woher kommen die hohen Zahlen? Gute Frage! (PS 828) |
| VS30 | Da der Schnelltest positiv war, konnte ich mich auf das ausstehende Ergebnis des PCR-Tests einstellen. (PS 285) |
| VS31 | Unsere Hausärztin führt Schnelltests durch. Somit war das Ergebnis innerhalb 2 min klar. Das wurde am nächsten Tag von Labor bestätigt. (OS 30) |
| VS32 | 1. Test Klinik PCR negativ 12.11., 2. Test Schnelltest Hausarzt 16.11. positiv Da der Schnelltest sofort positiv war > habe ich sofort alle Kontaktpersonen informiert, und in Quarantäne begeben. (PS 918) |
| **Interviews QUAL** | |
| **Accessing diagnosis** | |
| VS33 | Dann habe ich mich, äh, ja, habe ich, äh, dann einen Test gemacht in (andere Großstadt) am Flughafen, weil, äh, die Testzentren alle irgendwie in (Bundesland) etwas noch unstrukturiert waren. (15 NL) |
| VS34 | erstmal hat es irre lange gedauert, bis ich überhaupt irgendwie jemanden da an die Strippe kriegte, (…) dann wurde mir gesagt, ähm, aber, so Termine vergeben sie nicht, da müsste ich am nächsten Tag zum Hausarzt gehen. Und der Hausarzt war - natürlich - in Herbstferien. Dann musste ich bei einem Vertreter einen Termin kriegen (12 NL) |
| VS35 | es war Wochenende, natürlich an einem Samstag, wollte, haben wir beim ärztlichen Notdienst halt angerufen, gefragt, wie es aussieht. Ja, ob ich denn hoch Fieber hätte - ich habe aber nicht soo wahnsinnig hoch Fieber gehabt, nur so 38 Grad gerade so, und dann hieß es Nö, sie würden nicht testen, ich soll am Montag zum Hausarzt gehen. (9 NL) |
| VS36 | Die Symptome waren eindeutig. Also ich bin Mikrobiologe, ich verstehe was vom Fach, also das war für mich alles eindeutig gewesen. (…) man fällt durch dieses Raster! Also das war, das waren noch nicht gut ausgearbeitete Pläne oder Anweisungen, eh, Richtlinien, also man ist einfach durch dieses Raster durchgefallen. (4 LK) |
| VS37 | Der hat gleich was aufgebaut, ein Zelt, ein Zelt vor der Praxis im Garten. Dass man nicht in die Praxis reinmusste. Und das ging eigentlich problemlos. (2 LK) |
| VS38 | Und dann, ja, wurde halt dieser, sollte ich diesen Abstrich eben selbst machen, durchs Fenster, alles gesichert und so, also das war wirklich auch gut vorbereitet. (3 LK) |
| VS39 | fand ich das sehr, sehr, toll, dass mich die Hausärztin abends um kurz vor 10 noch angerufen hat, mir das Testergebnis quasi mitgeteilt hat und eben wie gesagt mir son paar kurze to dos mit auf den Weg gegeben hat. (14 LK) |
| VS40 | Am nächsten Tag hat die Ärztin mich dann angerufen, hat gesagt, ja jetzt, ehm, habe sie auch so Informationen, dass das vielleicht irgendwas damit zu tun haben könnte, wenn man nichts riecht und nichts schmeckt, ich sollte doch mal vorbeikommen zum Testen. Genau. Und da waren ja aber bereits zehn Tage vergangen. (6 LK) |
| VS41 | Als Hausarzt kann ich ja nicht sagen, Corona machen wir nicht! Das geht nicht, aber das hat diese Praxis so gehandhabt. (11 LK) |
| VS42 | und dann der übliche Alarm halt. Gesundheitsamt informieren, da Kontakt aufnehmen, Arbeitgeber informieren, und da ist man erst mal ein, zwei Tage beschäftigt, bis man das alles organisiert hat, und, ja, auch den ersten Schreck überwunden hat. (11 LK) |
| VS43 | hundert Millionen gefühlte Anrufe, und haben es dann endlich geschafft, dass dann an dem Donnerstagabend der Bereitschaftsdienst da um nachts um elf kam und die Jungs da vor der Haustür getestet hat. (…) Und, naja, alle positiv. Und dann auf einmal wurde die Klasse von meinem einen Sohn komplett in Quarantäne geschickt. (…) Weil einfach es keiner geschafft hat, unsre Kinder zu testen und wir nicht in der Lage waren, irgendwo hin zu fahren. (22 LK) |
| VS44 | ich habe es als sehr, also als schwierig erlebt, dass meine Kontaktpersonen alle gar nicht getestet wurden (…) Zum Beispiel mein Freund, der war dann bei seiner Familie und wurde erst nicht getestet (…) musste er dann letztendlich eine ganze Woche länger in Quarantäne und das war ein totales Heckmeck. (2 NL) |
| VS45 | Ja und dann hat sie noch gesagt, da würde noch jemand anrufen vom Gesundheitsamt und einen Abstrich machen, aber das war dann auch, mehrere Tage kam keine Rückmeldung gewesen, die waren erstmal überlastet. (2 NL) |
| VS46 | Also das Gesundheitsamt bei uns war jetzt völlig überfordert. (…) dann sagt die, ja ich soll ihr dann die Liste schicken mit den Kontakten, aber sie werden sowieso niemanden anrufen. Weil sie gar nicht die Zeit dazu haben. (6 NL) |
| VS47 | Und dann habe ich auch festgestellt, dass die Informationen da nicht weitergegeben worden sind. Und immer wieder ein anderer Ansprechpartner (…) ich habe an mehreren Tagen hintereinander das Gleiche beantworten müssen. (4 LK) |
| VS48 | Und da habe ich dann bei dem Telefonat mit dem Gesundheitsamt auch schon wieder so ein bisschen das Vertrauen verloren. Ehm, weil man einfach drei Ansprechpartner hat, und drei unterschiedliche Aussagen bekommt. (14 LK) |
| VS49 | und diese absurde Liste der Kontakte, die man handschriftlich ausfüllt und wieder per Post zurückschicken muss. Wo ich gedacht hab, ja, hm, dann wundert es mich nicht, dass die Kontaktnachverfolgung nicht funktioniert. (23 LK) |
| **Emotional response** | |
| VS50 | Und, ich habe es eigentlich, wenn es im Fernsehen oder in den Nachrichten so gebracht wurde, dass eigentlich viel im Privaten passiert, also das konnte ich mir nicht erklären, ich konnte es nicht glauben. Dann hat es mich selber erwischt. (7 NL) |
| VS51 | Und dann haben wir erstmal gesagt, hm, Corona, nein kann nicht sein, wir sind ja alle fit und gesund (…)ich habe sowas nicht, ich habe kein Asthma, nichts, ich bin normal kerngesund. (19 NL) |
| VS52 | da hatte ich auch bis dahin überhaupt nicht daran gedacht oder darüber nachgedacht, äh, dass da irgendwas nicht stimmen könnte. Es war wirklich wie, wie ein Schlag ins Gesicht. (1 NL) |
| VS53 | wie wir es erfahren haben, dann waren wir erstmal da gesessen, und erst mal weiß man gar nicht: Ja und jetzt, wie geht das jetzt weiter? Ne. Und, also an dem Abend, wo wir- als wirs erfahren haben, das war einfach wirklich furchtbar für uns, muss ich sagen. (17 LK) |
| VS54 | es hat mich jetzt nicht irgendwie umgehauen oder so, sicher hat man auch schon im Fernsehen immer die Bilder gesehen von der Intensivstation und so, aber sagema, ich hab mich jetzt nicht total geängstigt oder so. Ich hab damit irgendwie gelebt und hab halt gedacht, naja, mal abwarten die Entwicklung. (7 LK) |
| VS55 | die Gewissheit- also das hat mir wahnsinnig geholfen, das ist es jetzt. Man kann nichts machen. (23 LK) |
| VS56 | Dann zu der mentalen Geschichte: Nachdem, ehm, des- der Test positiv war, dann denkste natürlich nach, was war dann, ehm, in, in der Zeit? Ehm, Wo man sich eben, eh, eh, oder wo man des hätte weitergeben können. (24 LK) |
| VS57 | Hatte das positive Testergebnis. Wo ich mir natürlich gedacht hab, hmm, so, jetzt bist du da 5 Tage lang quasi wahrscheinlich infektiös gewesen und rumgelaufen! (23 LK) |
| VS58 | ja da ist mir schon irgendwie ein bisschen der Boden unter den Füßen weggezogen worden, weil, also ich bin bis zu dem Zeitpunkt davon ausgegangen, dass es sicherlich nichts ist, aber ... es war dann einfach eher die, ja, psychische Belastung, wo ich mir dann gedacht hab, mein Gott, jetzt war ich da in der Arbeit, ich war hier, ich war hier und ich war da. (6 NL) |
| **Illness experience** | |
| **SURVEY QUAL** | |
| **Disease progression and symptoms** | |
| VS59 | Aufgrund der Symptome wäre ich "normalerweise" nicht zum Arzt! (PS 100) |
| VS60 | Die Erkrankung mit Covid-19 war für mich die schwerste Erkrankung, ich hatte noch nie Todesangst. Selbst das Umdrehen im Bett war ein richtiger Kraftakt. (OS 68) |
| VS61 | Ich empfand die Erkrankung und den Verlauf schlimmer als meine Chemo die ich damals 2012 bekommen habe. (PS 514) |
| VS62 | In der Zeit im Krankenhaus ging es mir so schlecht, dass ich dachte ersterben zu müssen. ich hatte 10 Tage lang 40 Grad Fieber, obwohl ich mindestens 7 Tage 8 Tabletten Paracetamol genommen hatte. 8…) Nach dem Krankenhausaufenthalt hatte ich wochenlang Schlafprobleme (PS 12) |
| VS63 | Die anfänglichen Kopf-und Gliederschmerzen waren extrem stark, wusste nicht, dass ein Körper so wehtun kann. Keine kontinuierliche Besserung...mal 1 Tag besser, danach wieder schlechter, vorwärts-rückwärts, nach 1 Woche Husten und im allgemeinen sehr schwach! (PS 278) |
| VS64 | Die Kopfschmerzen waren sehr, sehr heftig. Ich habe mit Migräne zu kämpfen, aber diese Kopfschmerzen waren 10-fach stärker. Der komplette Geruchs- und Geschmacksverlust war sehr beängstigend. Die Gliederschmerzen und diese Kraftlosigkeit waren extrem stark. (PS 265) |
| VS65 | Ich hatte zum Mitte der Erkrankung circa 3-4 Tage wo ich unter extrem starken Husten gelitten habe und ich mich teilweise erbrechen musste, aufgrund der körperlichen Anstrengung. (OS 2) |
| VS66 | Mein Hauptproblem war unsagbare Mattigkeit. Konnte mich nur Schritt für Schritt fortbewegen, wie ein Mann mit 95 Jahren. (PS 191) |
| VS67 | Enorme Erschöpfungserscheinung, so noch nicht erlebt. (…) Relativ lange Dauer (über zwei Wochen), bis man sich wieder in der Lage fühlte, auch mal wieder raus zu gehen o.ä., bzw. den Körper und Kreislauf wieder in Schwung zu bringen. (OS 65) |
| VS68 | Die akute Erkrankungsphase habe ich als sehr belastend erlebt, aufgrund der vielen Symptome und der Tatsache, dass immer wieder neue Symptome dazukommen. (PS 939) |
| VS69 | Wechsel der Symptome, z.B. relatives Wohlbefinden > schlagartige Müdigkeit. (PS 779) |
| The Mental Burden caused by COVID-19 | |
| VS70 | Zunahme der Beschwerden, das hat mir wahnsinnig Angst gemacht, "was kommt noch", Kopfkino> Intensivstation (PS 220) |
| VS71 | Panikattacken in Verbindung mit nächtlichem Herzrasen; Angst vor Spätschäden (PS 775) |
| VS72 | Es war immer die Angst dabei. (PS 238) |
| VS73 | Auch die medial und politisch geschürte Angst vor einem schlimmen Verlauf war belastend. (PS 635) |
| VS74 | Ich gehöre wegen Vorerkrankung und Alter zur Risikogruppe, mein Mann ebenfalls, deshalb war die Sorge groß! (PS 508) |
| VS75 | Die zwei Wochen Quarantäne in der kleinen 2 Zimmer-Wohnung ohne Garten und Balkon im ersten Stock war die Hölle. 14 Tage ohne eine persönliche Begegnung war nicht schön, und wünsche ich niemandem. Alleine in der Wohnung, die Mutter mit Covid in einer anderen Wohnung alleine. (PS 868) |
| VS76 | Habe mich alleine gefühlt, da ich auch alleine wohne!  Habe eine 90jährige Mutter die ich angesteckt habe, da hab ich mich völlig überfordert gefühlt. Musste im Krankenhaus 4 Wochen alleine bleiben ist eine furchtbare Erfahrung und kein Besuch einfach schlimm!!! Hab keine Hilfe für meine Mutter war überhaupt nicht zu kriegen bis ich aus Not den Notdienst gerufen habe. (PS 566) |
| VS77 | Die Tatsache, dass man mit den Beschwerden alleine ist, da ja Quarantäne, ist belastend und schwierig zu ertragen. (PS 939) |
| **Access to health care** | |
| VS78 | Ich wurde nach dem Test niemals untersucht (auf Lungenentzündung etc). (PS 363) |
| VS79 | Ich persönlich habe die Erkrankung als sehr schlimm empfunden, das schlimmste aber ist, dass du zu keinem Arzt in die Praxis gehen darfst, bei Atemnot gehen Sie ins Krankenhaus hat es geheißen. (PS 303) |
| VS80 | Eine Information durch das GA erfolgte zu keiner Zeit! GA telefonisch nicht erreichbar. Zig mal nach 15 min. aus der Warteschleife "geflogen"! (PS 658) |
| VS81 | Ich fühlte mich mit Covid und in der Quarantäne allein gelassen. (OS 64) |
| VS82 | Ich habe mich allein gelassen gefühlt, da es keinen Ansprechpartner gab bei Symptomverschlechterung oder allgemeinen Fragen. (PS 104) |
| VS83 | Ich habe mich im März sehr alleingelassen gefühlt. Keiner hat einen untersucht. Keiner hat einem geholfen. Man wurde eingesperrt und man musste zusehen wie man damit klar kommt. Ich war glücklich, dass mich meine Tochter 21 Krankenschwester zu Hause so gut versorgt hat. Da ich alleinerziehend bin mit 3 Kindern, ein Risikokind absolut kein Geschenk. (PS 84) |
| VS84 | Während der Erkrankung gab es keine Unterstützung, weder vom Gesundheitsamt noch vom Hausarzt. Konnte nur telefonisch ein Rezept vom Hausarzt gegen Husten anfordern. Es fanden leider keine Gespräche statt. Ich war sehr hilflos in der Zeit. Untersuchung auf Lungenentzündung fand nicht statt. (PS 62) |
| VS85 | Keine Betreuung während der Krankheitsphase (danach auch nicht). Unsicherheit. (PS 102) |
| VS86 | Erschreckend war die Ratlosigkeit beim Anruf der 116117, als bei mir abends die ersten Symptome auftraten. (PS 1001) |
| VS87 | Ärzte erwecken den Eindruck über Unwissenheit durch mangelnde Erfahrung oder zu wenig Weiterbildung (OS 45) |
| VS88 | Es erfolgte keine Betreuung. Der Hausarzt hat an das Gesundheitsamt verwiesen, dieses hat sich jedoch erst nach Ablauf der Quarantäne gemeldet, da mein positiver Befund wohl nicht beim Amt ankam. (OS 88) |
| VS89 | Ich fühlte mich nicht gut betreut. GA und Hausarzt haben unregelmäßig angerufen und unterschiedliche Informationen weitergegeben (PS 616) |
| VS90 | Wurde von 2 Gesundheitsämtern betreut (XX1 wegen Arbeitsstelle, XX2 wegen Wohnort). Jedes Gesundheitsamt gab andere Informationen bezüglich neuen Tests, Arbeitsbeginn, Quarantäne, usw. Das war alles sehr verwirrend. (PS 541) |
| VS91 | Das Gesundheitsamt war sehr unorganisiert. Sie hatten keine EDV-Unterstützung und alles nur von Hand gemacht. Deswegen Mehrfachanrufe von Amtsärzten, die das Gleiche fragten... Es war alles chaotisch! Wir mussten um die Durchführung von Tests betteln, gerade weil wir keine Symptome hatten. (PS 610) |
| VS92 | Betreuung während der Quarantäne seitens des Gesundheitsamts war super. (PS 72) |
| VS93 | es war keine Betreuung erforderlich (PS 657) |
| VS94 | Der tägliche Telefonkontakt mit meiner Hausärztin tat seelisch so gut!!!! (PS 659) |
| VS95 | In dieser Zeit gab es viele Höhen und Tiefen. Und dank meiner Familie, Freunde und meinen tollen Hausarzt habe ich diese nicht ganz einfache Zeit überstanden. (PS 494) |
| **Interviews QUAL** | |
| **Disease course and progression** | |
| VS96 | ich hatte halt wirklich so stechende Kopfschmerzen wie noch nie in meinem Leben. (3 LK) |
| VS97 | Und hab dann halt eben auch ganz massive Probleme bekommen wirklich mit Muskeln, ne, also mit dem Muskelabbau einfach, also wirklich richtig Schmerzen, dass ich wirklich gedacht hab, ich kann des so nimmer! Des geht einfach net, des halt ich gar net aus. (20 LK) |
| VS98 | Diese Mattigkeit (…) eigentlich habe ich Durst, mal aufstehen, was zu Trinken zu holen aber viel zu große Anstrengung. (23 LK) |
| VS99 | die drei Wochen waren so furchtbar! (…) wir haben kein Fernsehen geguckt, wir haben kein Radio gehört, wir sind an kein Telefon gegangen, ich habe keine Whatsapp geschrieben. Nichts, null. Wir haben nur hier gelegen und haben geschlafen (…) und haben zum Fenster rausgeschaut. (6 NL) |
| VS100 | hohem Fieber, mit Herzrasen und mit Husten (…) ich habe irgendwie so alles mitgenommen, so gastrointestinal, Schmerzen, Erschöpfung, alles. (…)17 Tage war ich in der Quarantäne und vielleicht, sage ich jetzt mal, drei Wochen diese Akutsymptome. (8 NL) |
| VS101 | dann habe ich Fieber gekriegt, über 10 Tage lang immer 39. Dann ganz schlimme Nervenschmerzen, Trigeminusschmerzen, äh, Nervenendschmerzen am Rücken, an den Armen, (…) das war ganz schlimm. Dann, Magen-Darm, Erschöpfungszustände, äh, Übelkeit, Kurzatmigkeit, ich konnte keine Treppe mehr hochlaufen ohne dass ich mich wieder hinsetzen oder hinlegen musste. Müdigkeit auch, ich, wo ich mich hingesetzt habe, bin ich eingeschlafen. (10 NL) |
| VS102 | immer das Gefühl gehabt, auf meinen Brustkorb, da drücken irgendwie so zwei, drei Kilo drauf. (…) Also ich musste mich so richtig anstrengen, dass ich einatmen konnte. Und dann nachts habe ich mich dann irgendwie ein, zwei Mal erwischt, wie ich aufgekommen, aufgewacht und habe so nach Luft geschnappt (…) dann konnte ich nicht mehr schlafen, dann hatte ich Angst, dass ich irgendwie ersticke oder was weiß ich, wenn ich da wegschlafe. (7 NL) |
| VS103 | bin ich halt aufgewacht und habe gemerkt, kriegst schlechter Luft. Will aber auf gar keinen Fall von Atemnot sprechen, sondern eher von Kurzatmigkeit - man hat das Gefühl, nicht mehr durchatmen zu können. Das war so irgendwie da (atmet kurz demonstrativ ein), und dann war Schluss, ja? Und, ähm, muss dazu sagen, dann kriegt man richtig Panik, weil man ja sozusagen auch immer die anderen Sachen hört und denkt, Mensch, denkste, jetzt hast du es über den Berg, aber - wohl doch nicht. (11 NL) |
| VS104 | wahnsinnige Halluzinationen und dergleichen. (…) ich habe alles Mögliche gesehen, tagsüber. Und jeder Pfleger, der mein Zimmer betreten hat, oder auch die Ärzte, hatte ich immer den Eindruck, sie möchten mich umbringen. (16 LK) |
| VS105 | Wo des so eine, ja, eine göttliche Ruhe war. Ich mich selber auch gar nicht gespürt hab. Also ich habe nichts gesehen, nix, es war einfach nur n Gefühl, wo ich mich so richtig geborgen gefühlt hab. Und da habe ich mir gedacht, na des ist jetzt der Tod. Und eigentlich, schöner kann man gar nicht sterben, wenn die Leute wüssten, wie toll des ist, dass man keine Angst haben muss, und keine Schmerzen empfindet. (16 LK) |
| VS106 | so ein Zeitraum, wo ich gemerkt habe, dass ich … ja wieder von meinem Pegel her näher an der Reizschwelle eh, eh, entlangbewege, wo es dann auch mal .(…)Ja, zu ner kleinen Episode dann kommt. (11 LK) |
| VS107 | ich hab auch noch Parkinson. Und während den – während – ja, während dem Virus, also, wo ich halt auch Fieber hatte, da war so mein Gang ziemlich langsam. Also das hat mir irgendwie zugesetzt. Trotz meinen Medikamenten. (10 LK) |
| VS108 | dann ist mir der Blutdruck, weil ich meine Medikamente ja weiter genommen habe, äh, völlig in den Keller gerasselt, sodass ich dann mich auf meinem Fliesenboden im Badezimmer wiedergefunden habe (…) Und hatte mir dann drei Zähne ausgebrochen, als ich wieder zu mir kam. (4 NL) |
| VS109 | bin ich in der Nacht, als ich aufgestanden bin, zwei mal bewusstlos geworden und bin umgekippt. (…) Was mich schon erstaunt hat, war diese Geschwindigkeit, mit der sich die Symptome dann bei mir breitgemacht haben. (19 LK) |
| VS110 | hinzu kam ja dieses rapide, das rapide, eh, Verschlechtern der Situation. (…) Ich muss ja sagen, das sind ja auch sehr, sehr seltsame Symptome aufgetreten. Also ich hatte Schmerzen gehabt in der Brust, also im Herzbereich, ich hatte Schmerzen in der Lunge. (4 LK) |
| VS111 | also bei den 6 Personen waren es auch 6 verschiedene Symptome. (22 LK) |
| VS112 | ein Auf und ein Ab. Es war nicht wie einer normalen Erkältung, sondern ich hatte zwischendrin Phasen, wo es mir gut ging, wo ich gedacht habe, oh, ich würde jetzt lieber gerne arbeiten gehen, und am nächsten Tag schlagartig ging es mir dann wieder schlechter. Das war, wie so Schübe habe ich das empfunden. (16 NL) |
| VS113 | diese Ungewissheit – Wie verläuft das jetzt bei mir? Kann man da jetzt von Tag zu Tag was sagen, oder, oder, ähm, ja, gibt es da gar keine Hinweise? Und dann liest man natürlich dummerweise auch viel, auch viel im Internet, und da gibt es natürlich alles, ne? Also da gibt es, ähm, am zweiten Tag schon ganz schlimm, und dann gibt es natürlich auch erst in der dritten Woche so richtig schlimm, also das hilft einem natürlich auch nicht weiter. (1 NL) |
| VS114 | immer im Hinterköpfchen zu haben, das kann ganz schlimm ausgehen, ist nicht - ist nicht förderlich. Und ich finde das auch nicht in Ordnung vom Staat, dass der das so kommuniziert, weil das Erste, was man im Radio hört, wie viele an Corona gestorben sind. (11 NL) |
| VS115 | Aber ich muss schon sagen, wenn man natürlich so viel Zeit hat, und auch so viel Zeit hat, nachzudenken, empfindet man seinen Körper natürlich irgendwie schon auch viel sensibler, und viel, ähm, präziser, und man, man achtet viel mehr darauf, also, was mir vielleicht im Alltag so gar nicht aufgefallen wäre. (1 NL) |
| VS116 | Das geht mir eher an die Nieren, dass die des mitmachen mussten, wie mir selber, so, weil ich bin ja noch da, ne. (…) mein Mann war schon sehr, sehr mitgenommen. (16 LK) |
| VS117 | das war schon bitter für meine Frau, dass Sie gedacht hat, dass sie gedacht hat, sie sieht mich nicht mehr. (19 LK) |
| VS118 | Ich weiß noch das Letzte, was ich zu meiner Frau per SMS geschrieben hab, das ging noch, an dem Tag, an dem ich eingeliefert worden bin, die letzte Nacht, wo ich ihr geschrieben hab, dass ich sie liebe und, über alles, und an sie denke, und dass- das sind sehr, sehr tiefe Emotionen. (19 LK) |
| VS119 | der Arzt meinte, der Hausarzt, äh, wenn das jetzt damit nicht besser wird, müssen wir halt eine Aufnahme im Krankenhaus überlegen. Und, da ich schon wusste, dass das meistens nicht so gut endet, wenn man beatmet wird, wollte ich das halt um, also um Himmels willen nicht. (15 LK) |
| VS120 | die Ärzte hatten auch empfohlen, ob wir nicht ins Krankenhaus gehen wollten, äh, mit unserer Lungenentzündung, äh, die wir beide hatten (…) wir waren beide der Meinung, nein, zusätzliche, ähm, Keime sich da einzufangen im Krankenhaus, äh, bringt nichts. (3 NL) |
| **Living situation during isolation** | |
| VS121 | wenn ich das noch mal kriegen sollte, habe ich eigentlich den meisten Schiss vor der Isolation wieder. (15 LK) |
| VS122 | also es war schon bitter. Letztendlich war ich ja fast fünf Wochen in Quarantäne. (22 LK) |
| VS123 | Und war dann auch frustriert, und dann vor allem, weil des mit dem Ergebnis so lang gedauert hat, bis des da war. Und, und halt dann, weil die Quarantäne dann auch verlegt wurde, und, ja, also, des war dann, für die war des net spaßig, (…) des war für mich auch belastend irgendwie. (21 LK) |
| VS124 | Und dann war nicht mal klar, sind meine Symptome 48h vor meinem Quarantäneende wirklich durch? Also bin ich dann frei? (…) Und da habe ich die ganze Zeit so im Kopf, okay, wenn ich da jetzt noch ne Woche- 28 Tage Quarantäne, nee! Nee! (13 LK) |
| VS125 | ich, äh, habe ja das Glück, dass ich ein Haus habe und einen großen Garten, und dass meine Kinder auch immer in den Garten konnten zum spielen und so, also, da geht es uns ja sehr gut. (17 NL) |
| VS126 | Also wir haben einen großen Garten, das Wetter war schön, also da hatte ich überhaupt keine Probleme damit. (5 LK) |
| VS127 | Ich konnte zumindest in den Garten raus und mit meiner Tochter kurz mal in einem guten Moment, des war am Sonntag in am guten Moment, da ham mer denkt es geht wieder aufwärts – da a weng, mit ihr dann a weng Fußball hin und her gespielt. Des wor halt a ganz an anderer...ja, Wohlfühlmoment als wenn mer so okay, ich bin die ganze Zeit irgendwo eingesperrt. (9 LK) |
| VS128 | mir gings…prächtig. Ich habe die Zeit genutzt, um mein halbes Haus zu renovieren. (11 LK) |
| VS129 | Wir hatten was zu tun. Das war die Hauptsache. Ich glaub, wenn wir gar nichts zu tun gehabt hätten, das wäre dramatischer gewesen. (15 LK) |
| VS130 | es ga- gibt Momente, muss ich ehrlich sagen, wo ich mir gedacht hab, da hätte ich doch ins Krankenhaus gemusst. Um einfach, dass die Familie wahrnimmt, hallo, mir geht’s nicht gut. (…) Man kann des net da, Auszeit nehmen. Also, nicht, wenn mer halt so viele Kinder hat einfach. (22 LK) |
| VS131 | Dann musst du ständig die Namen durchgeben, und das mal sechs. Und das Geburtsdatum mal sechs. Dann die Quarantäne. Jeder hat n andern Tag Quarantäne gehabt. (…). Und das war eben diese eine Woche, wo es halt grad echt so schlecht ging. (22 LK) |
| VS132 | mein Kind war ein halbes Jahr alt und als Mutter muss man dann halt funktionieren (lacht), aber irgendwie war es ganz schön anstrengend, ich hatte ganz schöne Gliederschmerzen. (1 LK) |
| VS133 | drei Wochen, wo wir nicht rausdurften, eh, war schon extrem anstrengend. Weil man sitzt wirklich zu viert auf, auf engerem Raum. Eh, man kann sich nich groß ausm Weg gehen, und irgendwann fällt einem auch die Decke aufn Kopf. Und, und, eh, dann auch mit den Kindern, die dann irgendwann, eh, ja auch, eh, rammdösig werden, eh, streiten sich, also, des, des Potenzial steigt dann schon. (18 LK) |
| VS134 | Und dann musste halt die Familie, und halt auch die Hormone und die Pubertät so in einen Hut bringen, des ist- also, es war schon ne ganz blöde Zeit. (22 LK) |
| VS135 | Ja, ich musste dann mit der Laune meines Mannes klarkommen. (1 LK) |
| VS136 | die Trennung von der Tochter. Also des- für die wars wirklich schwer. Die war teilweise wirklich vor der Tür gesessen und hat geweint und konnt halt net reinkommen (17 LK) |
| VS137 | zum Glück sind wir familiär da wirklich, also gut, äh, passt des sehr gut zusammen. Auch mit meinem Mann und so, dass mer da gut durchkommen sind. Also, und auch die Kinder. Also, des ham mer auch im Nachhinein jetzt immer wieder gesagt, dass es trotz allem, wie des alles war, trotzdem, dass sie alle super mitgemacht haben und dass es wirklich gut geklappt hat. (17 LK) |
| VS138 | diese Isolation ist glaube ich mit das Schlimmste an der Erkrankung selber. Also gar nicht mal nur das Medizinische, sondern tatsächlich, dass man einfach so allein ist. (1 NL) |
| VS139 | und dann meine Mutti am Telefon, die dann auch äh, völlig depressiv war und ständig geweint hat, und, und, wir konnten ja auch nicht. Was wollte ich denn machen? Wir haben uns Weihnachten nicht gesehen, wir haben uns Silvester nicht gesehen. (6 NL) |
| VS140 | das war für mich nicht so wirklich einfach, also gerade psychisch, weil ich eben 10 Tage lang komplett alleine eben in dieser Einliegerwohnung war. (2 NL) |
| VS141 | Dann, eh, und die Enkelkinder, keiner ist gekommen, alle nur so mit Abstand. Ich hab 10 Enkelkinder (lacht), ja. Und alle nur mit Abstand, und, ich, das war… (8 LK) |
| VS142 | das familiäre und nachbarschaftliche Netzwerk, die dann auch sich angeboten haben, einkaufen zu gehen und so. Also Das hat einem schon gut getan, dass man wusste, man hängt da nicht allein drin. (5 LK) |
| VS143 | das ist ja das Schlimme, wenn Sie in einer Quarantäne sind: Ihnen kann niemand helfen. Sie, also das heißt, unser, unsere Freunde haben uns zwar versorgt, mit Lebensmitteln, (…) aber ich war drei Wochen nicht in der Lage, irgendwas zu kochen. (6 NL) |
| VS144 | mer wird a weng gemieden, also eh, weil ja die ganzen Leute net umgehen haben können, so ungefähr. Was ist jetzt dieses Neue, und die da, die haben Corona, da müss mer schnell weitergehen, ne. Hat mer zwar nie was gsogt, aber mer hat am Anfang hat mer, von a paar Menschen hat mer schon gemerkt, aha, des is, eh, mer will kein Kontakt. (9 LK) |
| VS145 | Ende Oktober, da war ich, ja, war in unserem nähere Freundes- und Verwandtschaftskreis, war ich der erste und der einzige, so (…) mer fühlt sich schon irgendwie, ehm, so bissl, ja, sag mal, ich nutz jetzt den Begriff aussätzig, aber, ja, vielleicht ist der ja bissl extrem gewählt, aber, ja, man wird halt dann doch, ehm, ja, gemieden und, und mhh, gefragt, ehm, die Leut interessieren sich dafür und so weiter und so fort, ja. (21 LK) |
| VS146 | dann die Tatsache, dass ich im Krankenhaus war, und die Sanitäter mich dann dort abgesetzt haben, und im Rausgehen dann noch gesagt haben, so, jetzt können wir unseren Rettungswagen wieder stundenlang desinfizieren.. das hat mich eigentlich mehr getroffen, wie alles andere. (16 LK) |
| **Access to health care** | |
| VS147 | Und auch im Internet. Also überall, wo ich mich informiert hatte, das hieß immer, diese Hotline anrufen. Die Hotline und Gesundheitsamt. Weil die waren beide nicht erreichbar. (4 LK) |
| VS148 | irgendwann kommt man an einen Punkt, wo man einfach Angst bekommt. Wer hilft mir dann. Es war einfach unmöglich, da Hilfe zu bekommen (…) ich wusste nicht, wie schlimm wird es dann in der kommenden Nacht. Also, ist man dann überhaupt noch in der Lage aufzustehen, zu telefonieren, oder irgendwas zu machen? (…) Man merkt, es geht rapide schlechter, und es ist keine Hilfe da. (4 LK) |
| VS149 | Diese Hilflosigkeit, das ist übelst. Das ist Wahnsinn. Und vor allen Dingen, unsere Freunde haben dann auch zu uns gesagt, was ist, was wäre denn jetzt gewesen, ihr hättet niemanden gehabt? Und bei euch wäre hier, ihr wärt hier umgefallen, das hätte gar keiner gemerkt! (...) Ne? Also das, das ist, das ist furchtbar! Und das ist das, was, was, äh mir auch sehr an die Psyche auch gegangen ist. (6 LK) |
| VS150 | wenn man das alles so alleine durchstehen muss, da fühlt man sich einfach so alleine und verlassen. Also das habe ich mich ja schon ein bisschen gefühlt. Weil, natürlich guckt einen niemand an. Ja? Und man hat die einzige Möglichkeit, dann in die Klinik zu gehen. Also, ich hatte während der Erkrankung sehr viel Angst. (8 NL) |
| VS151 | wir waren dann schon sehr hilflos hier zu Hause. Muss ich echt sagen. (7 LK) |
| VS152 | mein Kollege hat mir irgendwie, das Blut abgenommen und geguckt, ob ich irgendwie Heparin brauche oder irgendwas, und mein Sauerstoff ist nicht unter 93 gegangen. Sodass ich irgendwie - meine Tochter hat mir Infusionen gemacht, die ist MFA - ja, so. Das heißt, ich konnte es echt zu Hause machen, das ist natürlich die Ausnahme. (13 NL) |
| VS153 | In der Apotheke, wie ich war, ehm, die ist- hat mir auch noch mal gesagt, mit was einreiben kann was helfen gegen Husten. Also wirklich, ehm, hab mich da schon gut aufgehoben gefühlt. (23 LK) |
| VS154 | aber ich fühl mich do, sog i mal in der alternativen, äh, mit Osteopathie ganz gut aufgehoben (24 LK) |
| VS155 | Ebenfalls ist gut, ich hab hier zu Hause n Pulsoxymeter, wo wir hier zu Hause SpO2 messen konnten und Pulsfrequenz. Dass wir son bisschen eben selbst kontrollieren konnten, ob es da Probleme gibt, die wir so quasi aufn ersten Blick gar nicht sehen. (14 LK) |
| **Personal strength and resilience** | |
| VS156 | auch Physiotherapie studiert, das heißt, ich kann mich weitestgehend auch gut mit der Wissenschaft schon so- oder bin mit der Wissenschaft vertraut. (14 LK) |
| VS157 | mein Mann ist vor fünf Jahren neben mir im Auto gestorben beim Autofahren, an Herzinfarkt. (…) das hat mich so, äh, so geprägt, dass ich, dass ich solche Angstzustände gekriegt habe, wo er ständig aufgestanden, ans Fenster gegangen ist, nach Luft geschnappt hat. Ich habe, ich habe permanent Angstzustände hier gehabt. (…) ich habe so eine Angst gehabt, dass mir das nochmal jetzt passiert, einen geliebten Partner zu verlieren.(6 NL) |
| VS158 | Was bei mir noch, äh, dazukommt, ist, dass ich, äh, seit 2007, ähm, aufgrund von einem starken Burnout in psychologischer Behandlung bin, und mir das eigentlich jetzt auch psychisch sehr mitspielt. (6 NL) |
| VS159 | obwohl die Symptome nicht so dramatisch waren, oder eigentlich gar nicht dramatisch, habe ich natürlich die Nachrichten konsumiert. Und das 20 Stunden am Tag! (…) diese LKWs in Spanien, oder, oder Italien, wo sie die Leichen abtransportiert haben. (…)Dann kamen noch diese Nachrichten: “Ahh, es können später noch Symptome auftreten”, man wird dann auch irgendwie so hellhörig und hört das Gras wachsen, und … (seufzt) (11 LK) |
| VS160 | Ich bin da in solchen Situationen sehr pragmatisch. (…) ich habe mir gedacht, so schlimm wird’s schon net werden. Jetzt wartest erst mal in der Quarantäne, und wenn nicht, gehst Du halt ins Krankenhaus. (16 LK) |
| VS161 | Ich bin recht gefestigt so im, im Selbstbewusstsein, im Auftreten und so was. auch so durch die Tätigkeit als Teamleiter. (…) wenn man 30 Leute unter sich hat, eh, verträgt man bissl Druck.(…) man is nicht ganz so labil. Wenn jetzt so eine Sache über einen einbricht, dass man nicht grad so geschockt ist und eh, sich jetzt da so groß Gedanken macht. (18 LK) |
| **Feelings of guilt and worries** | |
| VS162 | wenn du dann positiv bist, des Gedankenkarrussell, Mensch, mit wem, wo, eh, was kommt in Frage, ist jemand anders krank geworden, ehm, des war eigentlich die, die für mich mental, eh, belastendste Situation. (24 LK) |
| VS163 | ich hatte Schuldgefühle, oder mir einfach selber den Vorwurf gemacht, ja, dass ich eben Menschen vielleicht angesteckt habe. Und, mei, sogar in meiner Familie (…) hoffentlich passiert da jetzt nichts, mit Eltern, die jetzt ja schon über siebzig. (…) Da wir, da ich in einer Patchworkfamilie leben, hat es dann natürlich auch den Vater meiner Kinder getroffen. Und der hat mir auch tatsächlich Vorwürfe gemacht, also (lacht) das war irgendwie, also, irgendwie bisschen belastend. (6 LK) |
| VS164 | Und die hat dann auch heute noch Beschwerden mit der Luft. Die hat das noch schlimmer getroffen gehabt wie mich. (…) Ja, das war auch sehr, sehr schuld- schuldbewusst (lacht), hab ich mich gefühlt, sehr schuldig. (8 LK) |
| VS165 | Das belastet mich natürlich, weil ich mir schon Sorgen mache, oder Gedanken mach, ja, weil er es eigentlich ja durch mich bekommen hat (…) und ich wünsch, dass des natürlich so schnell wie möglich alles, eh, eh, wieder, wieder weggeht und dass er dann, eh, wieder ganz normal fit wird. (24 LK) |
| VS166 | ich habe dann, äh, auch sehr Schlafstörungen gehabt - habe mich dann, also, na, äh, mein Partner, der hat ja so, so, schlimm mit der Lunge zu tun gehabt, der hatte 83% Sauerstoffsättigung, ist nicht ins Krankenaus. Der ist jede Nacht aufgestanden, ans Fenster, sodass ich kaum die ersten drei Wochen geschlafen habe, und das hat mir natürlich auch sehr mitgespielt. (6 NL) |

# S7: Self-reported COVID-19 symptoms during acute disease

# S8: STROBE Statement—checklist of items that should be included in reports of Cross-sectional studies

|  | **Item No.** | **Recommendation** | **Page  No.** | **Relevant text from manuscript** |
| --- | --- | --- | --- | --- |
| **Title and abstract** | 1 | (*a*) Indicate the study’s design with a commonly used term in the title or the abstract | 1 |  |
|  |  | (*b*) Provide in the abstract an informative and balanced summary of what was done and what was found | 1-2 |  |
| **Introduction** | | | |  |
| Background/rationale | 2 | Explain the scientific background and rationale for the investigation being reported | 2 |  |
| Objectives | 3 | State specific objectives, including any prespecified hypotheses | 2 |  |
| **Methods** | | | |  |
| Study design | 4 | Present key elements of study design early in the paper | 3 | 2.1 Study design, Figure 1 |
| Setting | 5 | Describe the setting, locations, and relevant dates, including periods of recruitment, exposure, follow-up, and data collection | 3-4 | 2.2. Study population and recruitment; 2.3. Data collection and analysis |
| Participants | 6 | (*a*)—Give the eligibility criteria, and the sources and methods of selection of participants | 3 | 2.2 Study population and recruitment |
| Variables | 7 | Clearly define all outcomes, exposures, predictors, potential confounders, and effect modifiers. Give diagnostic criteria, if applicable | 3-4 | 2.3. Data collection and analysis |
| Data sources/ measurement | 8* | For each variable of interest, give sources of data and details of methods of assessment (measurement). Describe comparability of assessment methods if there is more than one group | 4 | 2.3. Data collection and analysis; Suppl. file S3 |
| Bias | 9 | Describe any efforts to address potential sources of bias | 3-4, 11 | 2.1 Study design, 2.2. Study population and recruitment; 2.3. Data collection and analysis, 4.3. Strengths and limitations |
| Study size | 10 | Explain how the study size was arrived at | 3-4 | 2.2. Study population and recruitment; Figure 2 |

| Quantitative variables | | 11 | | Explain how quantitative variables were handled in the analyses. If applicable, describe which groupings were chosen and why | 3-4 | 2.3.1. Quantitative data |
| --- | --- | --- | --- | --- | --- | --- |
| Statistical methods | | 12 | | (*a*) Describe all statistical methods, including those used to control for confounding | 3-4 | 2.3.1. Quantitative data |
|  |  |  |  | (*b*) Describe any methods used to examine subgroups and interactions | 3-4 | 2.3.1. Quantitative data |
|  |  |  |  | (*c*) Explain how missing data were addressed |  | Figure 2, Table 1, Table 2 |
|  |  |  |  | (*d*) If applicable, describe analytical methods taking account of sampling strategy |  |  |
|  |  |  |  | (*e*) Describe any sensitivity analyses |  |  |
| **Results** | | | | | | |
| Participants | | 13* | | (a) Report numbers of individuals at each stage of study—eg numbers potentially eligible, examined for eligibility, confirmed eligible, included in the study, completing follow-up, and analysed |  | Figure 2 |
|  |  |  |  | (b) Give reasons for non-participation at each stage |  | Figure 2 (when applicable) |
|  |  |  |  | (c) Consider use of a flow diagram |  | Figure 2 |
| Descriptive data | | 14* | | (a) Give characteristics of study participants (eg demographic, clinical, social) and information on exposures and potential confounders |  | Table 1 |
|  |  |  |  | (b) Indicate number of participants with missing data for each variable of interest |  | Table 1, Table 2 |
| Outcome data | | 15* | | Report numbers of outcome events or summary measures |  | *NA* |
| Main results | | 16 | | (*a*) Give unadjusted estimates and, if applicable, confounder-adjusted estimates and their precision (eg, 95% confidence interval). Make clear which confounders were adjusted for and why they were included |  |  |
|  |  |  |  | (*b*) Report category boundaries when continuous variables were categorized |  | Table 1, Table 2 |
|  |  |  |  | (*c*) If relevant, consider translating estimates of relative risk into absolute risk for a meaningful time period |  |  |
| Other analyses | 17 | | Report other analyses done—eg analyses of subgroups and interactions, and sensitivity analyses | |  | Suppl. File S1: Triangulation protocol |
| **Discussion** | | | | | | |
| Key results | 18 | | Summarise key results with reference to study objectives | | 9 | 4.1 Summary of the main findings |
| Limitations | 19 | | Discuss limitations of the study, taking into account sources of potential bias or imprecision. Discuss both direction and magnitude of any potential bias | | 11 | 4.3 Strengths and limitations |
| Interpretation | 20 | | Give a cautious overall interpretation of results considering objectives, limitations, multiplicity of analyses, results from similar studies, and other relevant evidence | | 10-12 | 4.2 Comparison with existing literature; 4.4 Implications for research and practice |
| Generalisability | 21 | | Discuss the generalisability (external validity) of the study results | | 11 | 4.3 Strengths and limitations |
| **Other information** | | |  | | | |
| Funding | 22 | | Give the source of funding and the role of the funders for the present study and, if applicable, for the original study on which the present article is based | | 12 | Funding |

*Give information separately for cases and controls in case-control studies and, if applicable, for exposed and unexposed groups in cohort and cross-sectional studies.

**Note:** An Explanation and Elaboration article discusses each checklist item and gives methodological background and published examples of transparent reporting. The STROBE checklist is best used in conjunction with this article (freely available on the Web sites of PLoS Medicine at http://www.plosmedicine.org/, Annals of Internal Medicine at http://www.annals.org/, and Epidemiology at http://www.epidem.com/). Information on the STROBE Initiative is available at [www.strobe-statement.org](http://www.strobe-statement.org).

Cuschieri S. The STROBE guidelines. Saudi J Anaesth. 2019 Apr;13(Suppl 1):S31-S34. doi: 10.4103/sja.SJA_543_18. PMID: 30930717; PMCID: PMC6398292.

1. **S9: COREQ consolidated criteria for reporting qualitative research checklist**

| **No** | **Item** | **Guide questions/description** | **Page; Additional Information** |
| --- | --- | --- | --- |
| **Domain 1: Research team and reflexivity** |  |  |  |
| Personal Characteristics |  |  |  |
| 1. | Interviewer/facilitator | Which author/s conducted the interview or focus group? | 4; 2.3.2. Qualitative data |
| 2. | Credentials | What were the researcher's credentials? *E.g. PhD, MD* | 4; 2.3.2. Qualitative data |
| 3. | Occupation | What was their occupation at the time of the study? | 4; 2.3.2. Qualitative data |
| 4. | Gender | Was the researcher male or female? | 3 female, 1 male |
| 5. | Experience and training | What experience or training did the researcher have? | 4; 2.3.2. Qualitative data |
| Relationship with participants |  |  |  |
| 6. | Relationship established | Was a relationship established prior to study commencement? | Contact via Email/ Phone and explanation of study, informed consent |
| 7. | Participant knowledge of the interviewer | What did the participants know about the researcher? e*.g. personal goals, reasons for doing the research* | Study Objectives; Study part of thesis |
| 8. | Interviewer characteristics | What characteristics were reported about the interviewer/facilitator? e.g. *Bias, assumptions, reasons and interests in the research topic* | Thesis student |
| **Domain 2: study design** |  |  |  |
| Theoretical framework |  |  |  |
| 9. | Methodological orientation and Theory | What methodological orientation was stated to underpin the study? *e.g. grounded theory, discourse analysis, ethnography, phenomenology, content analysis* | 4; 2.3.2. Qualitative data |
| Participant selection |  |  |  |
| 10. | Sampling | How were participants selected? *e.g. purposive, convenience, consecutive, snowball* | 3; 2.2. Study population and recruitment |
| 11. | Method of approach | How were participants approached? e*.g. face-to-face, telephone, mail, email* | 3; 2.2. Study population and recruitment |
| 12. | Sample size | How many participants were in the study? | 5; 3.1. Sociodemographic characteristics of the study population; |
| 13. | Non-participation | How many people refused to participate or dropped out? Reasons? | NA (participants directly contacted research team) |
| Setting |  |  |  |
| 14. | Setting of data collection | Where was the data collected? e*.g. home, clinic, workplace* | 4; (via telephone from workplace) |
| 15. | Presence of non-participants | Was anyone else present besides the participants and researchers? | SP (thesis supervisor) was present during first 2 interviews (this was explained to participants) |
| 16. | Description of sample | What are the important characteristics of the sample? *e.g. demographic data, date* | 5; 3.1. Sociodemographic characteristics of the study population; Suppl. File S4 |
| Data collection |  |  |  |
| 17. | Interview guide | Were questions, prompts, guides provided by the authors? Was it pilot tested? | 3-4; 2.2. Study population and recruitment; 2.3.2. Qualitative data;  Suppl. File S3 |
| 18. | Repeat interviews | Were repeat interviews carried out? If yes, how many? | No |
| 19. | Audio/visual recording | Did the research use audio or visual recording to collect the data? | 4; 2.3.2. Qualitative data |
| 20. | Field notes | Were field notes made during and/or after the interview or focus group? | Yes; Suppl. File S3 |
| 21. | Duration | What was the duration of the interviews or focus group? | 20-40 minutes |
| 22. | Data saturation | Was data saturation discussed? | 3-4; Suppl. File S3 |
| 23. | Transcripts returned | Were transcripts returned to participants for comment and/or correction? | No |
| **Domain 3: analysis and findings**z |  |  |  |
| Data analysis |  |  |  |
| 24. | Number of data coders | How many data coders coded the data? | 4; 2.3.2. Qualitative data |
| 25. | Description of the coding tree | Did authors provide a description of the coding tree? | 5-9; Table 3 represents coding tree |
| 26. | Derivation of themes | Were themes identified in advance or derived from the data? | 4; 2.3.2. Qualitative data |
| 27. | Software | What software, if applicable, was used to manage the data? | 4; 2.3.2. Qualitative data |
| 28. | Participant checking | Did participants provide feedback on the findings? | No; the findings were however discussed with COVID-19 patients and GPs |
| Reporting |  |  |  |
| 29. | Quotations presented | Were participant quotations presented to illustrate the themes / findings? Was each quotation identified? e*.g. participant number* | All themes and findings are illustrated with Verbatims in Suppl. S 5&6, incl. participants number (166 Verbatims) |
| 30. | Data and findings consistent | Was there consistency between the data presented and the findings? | 9-11; Table 3, Suppl. S5&6, Triangulation |
| 31. | Clarity of major themes | Were major themes clearly presented in the findings? | 5-9; Table 3; Suppl. S5&6 |
| 32. | Clarity of minor themes | Is there a description of diverse cases or discussion of minor themes? | 5-9; Table 3, Suppl. S1, S5-7 |

Tong A, Sainsbury P, Craig J. Consolidated criteria for reporting qualitative research (COREQ): a 32-item checklist for interviews and focus groups. Int J Qual Health Care. 2007 Dec;19(6):349-57. doi: 10.1093/intqhc/mzm042. Epub 2007 Sep 14. PMID: 17872937.

**
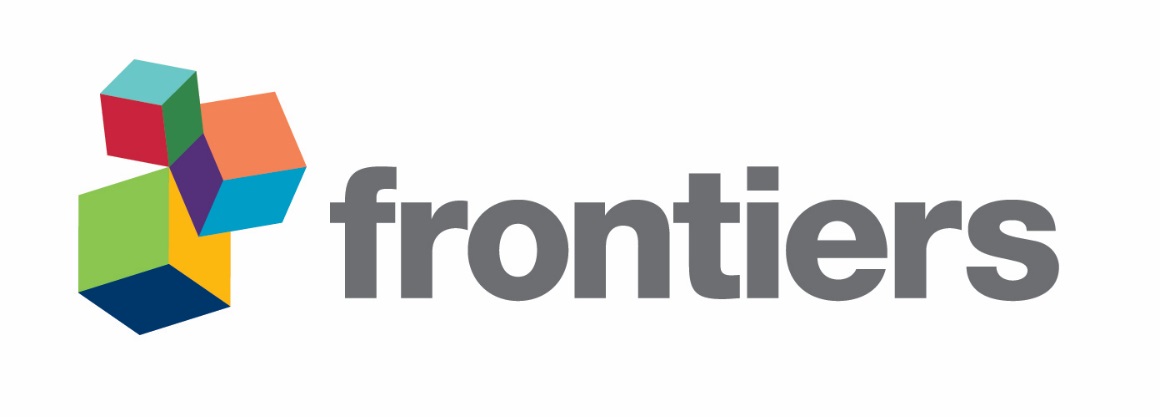
**
